# Supplementary material for: Confining donor conformation distributions for efficient thermally activated delayed fluorescence with fast spin-flipping
Source: Nat Commun. 2023 May 4;14:2564. doi: 10.1038/s41467-023-38197-y (PMC10160101; doi:10.1038/s41467-023-38197-y)
Supplement: Supplementary file 1 — Supplementary Information [file 41467_2023_38197_MOESM1_ESM.pdf]

Supplementary Information

Confining donor conformation distributions for efficient thermally activated delayed fluorescence with fast spin-flipping

Weidong Qiu[1], Denghui Liu[1], Mengke Li[1]*, Xinyi Cai[1], Zijian Chen[1], Yanmei He[1], Baoyan Liang[2], Xiaomei Peng[1], Zhenyang Qiao[1], Jiting Chen[1], Wei Li[1], Junrong Pu[1], Wentao Xie[1], Zhiheng Wang[1,2], Deli Li[1], Yiyang Gan[1], Yihang Jiao[1], Qing Gu[1] & Shi-Jian Su[1]*

1. State Key Laboratory of Luminescent Materials and Devices Institute of Polymer Optoelectronic Materials and Devices, South China University of Technology, Guangzhou 510640, P. R. China.
2. Ji Hua Laboratory, Foshan 528200, P. R. China.

* Corresponding authors, email: limk@scut.edu.cn, mssjsu@scut.edu.cn.

Supplementary Methods

^{1}H and ^{13}C NMR spectra were recorded on a Bruker NMR spectrometer operating at 400 and 150 MHz, respectively. High-resolution mass spectrometry was conducted on a Bruker maXis impact mass spectrometer under positive mode. MALDI-TOF mass spectrometry was conducted on a Waters SYNAPT G2-Si mass spectrometer. Thermogravimetric analyses (TGA) were performed on Netzsch TG 209 under nitrogen flow at a heating rate of 10 °C min^{-1}. The differential scanning calorimetry analysis was conducted using a NETZSCH (DSC-204) instrument with the rate of increasing temperature at 10 °C min^{-1} under nitrogen flushing. The HOMO energy levels were obtained from photoelectron yield measurement (AC-3) of the neat films, and the LUMO energy level can be calculated by the on-set of the absorption spectra. Single crystal of TBP-3aDMAc was obtained from slow diffusion of acetonitrile into its chloroform solution. For small molecule single crystal X-ray analysis, the data were collected on Rigaku XtaLAB P2000 FR-X with a rotating copper anode and a Pilatus 200 K detector at 150 K, and the structure was solved by the SHELXL package using the Olex 2.1.3 software. *P*-polarized angle-dependent light emissions of thin film were measured by Fluxim. The horizontal orientation ratio of the transition dipole moment in the emission layers and the optical power dissipation to the different optical modes in solution-process OLED were simulated by Setfos 5.1.

Synthesis of benzene-1,3,5-triyltris((4-(1,3,6,8-tetramethyl-9H-carbazol-9-yl)phenyl)methanone) (TBP-3MCz). 1,3,6,8-tetramethyl-9H-carbazole (1.41 g, 6.3 mmol) and TBP-Br (1.13 g, 1.5 mmol) were dissolved into 100 mL toluene in a 150 mL three-necked flask. The mixture was degassed for 15 minutes, then palladium(II) acetate ($Pd(OAc)_2$) (50 mg, 0.3 mmol), sodium *tert*-butoxide (tBuONa) (865 mg, 9 mmol) and tri(*tert*-butyl) phosphine ($P(t\text{-}Bu)_3$) (0.6 mL, 0.6 mmol) were added in the three-necked flask. And the mixture was degassed for 30 min. The mixture was heated to 110 °C and continually stirred for 12 h. After removing the solvent in vacuum, the mixture was partitioned between DCM and water. The combined organic layers were washed with brine, dried over anhydrous Mg_2SO_4 and concentrated in vacuo. Column chromatography of the residue solid (eluent: DCM/PE=1/1) afforded 1.2 g yellow solids (Yield: 78%). ^{1}H NMR (500 MHz, Chloroform-*d*) δ 8.57 (s, 3H), 8.03 – 7.96 (m, 6H), 7.76 (d, J = 1.8 Hz, 6H), 7.68 – 7.62 (m, 6H), 6.88 (d, J = 1.7 Hz, 6H), 2.49 (s, 18H), 1.88 (s, 18H). ^{13}C NMR (126 MHz, $CDCl_3$) δ 193.66, 147.63, 139.47, 138.20, 136.02, 134.16, 131.70, 130.43, 130.08, 130.01, 129.61, 124.58, 121.04, 121.00, 117.96, 77.27, 77.22, 77.02, 76.76, 21.09, 19.67, 19.63. HRMS calcd for $C_{75}H_{63}N_3O_3$ 1053.4869 (1076.4767, M+Na), found 1076.4762 [M+Na]. (Supplementary Fig. 28-30)

Synthesis of TBP-3aDMAc. Reaction of intermediate 10H-spiro[acridine-9,2'-adamantane] with TBP-Br following the same procedure for synthesis of TBP-3MCz generated the pure TBP-3aDMAc as a yellow power (Yield: 64%). ^{1}H NMR (500 MHz, Chloroform-*d*) δ 8.41 (s, 3H), 7.95 – 7.91 (m, 6H), 7.61 – 7.54 (m, 12H), 7.43 – 7.39 (m, 6H), 7.14 – 7.10 (m, 12H), 3.01 (d, J = 12.7 Hz, 6H), 2.93 (q, J = 2.8 Hz, 3H), 2.34 (q, J = 3.0 Hz, 3H), 1.99 – 1.93 (m, 6H), 1.85 (t, J = 3.4 Hz, 6H), 1.74 (d, J = 12.3 Hz, 3H), 1.63 – 1.53 (m, 6H), 1.49 – 1.38 (m, 13H). ^{13}C NMR (126 MHz, $CDCl_3$) δ 193.58, 147.04, 141.79, 141.02, 138.90, 133.02, 132.78, 129.10, 127.15, 125.01, 123.90, 123.39, 116.42, 77.27, 77.22, 77.02, 76.76, 48.45, 37.95, 35.30, 33.94, 33.48, 30.84, 29.33, 27.60. HRMS calcd for

$C_{93}H_{81}N_3O_3$ 1287.6278 (1310.6176, M+Na), found 1310.6170 [M+Na]. (Supplementary Fig. 31-33)

Synthesis of 2,4,6-tris(4-(1,3,6,8-tetramethyl-9H-carbazol-9-yl)phenyl)-1,3,5-triazine (TRZ-3MCz). Reaction of intermediate 1,3,6,8-tetramethyl-9H-carbazole with 2,4,6-tris(4-bromophenyl)-1,3,5-triazine following the same procedure for synthesis of TBP-3MCz generated the pure TRZ-3MCz as a yellow power (Yield: 82%). ^{1}H NMR (500 MHz, Chloroform-*d*) δ 9.02 – 8.92 (m, 6H), 7.79 (d, J = 1.8 Hz, 6H), 7.76 – 7.70 (m, 6H), 6.96 (d, J = 1.8 Hz, 6H), 2.51 (s, 18H), 1.97 (s, 18H). ^{13}C NMR (126 MHz, $CDCl_3$) δ 171.26, 146.88, 139.27, 136.03, 131.76, 130.32, 129.23, 128.98, 124.32, 121.14, 117.89, 77.27, 77.22, 77.01, 76.76, 21.11, 19.61. MALDI-TOF MS calcd for $C_{69}H_{60}N_6$ 973.3, found 973.7. (Supplementary Fig. 34-36)

Synthesis of tris(2,6-dimethyl-4-(1,3,6,8-tetramethyl-9H-carbazol-9-yl)phenyl) borane (TB-3MCz). Reaction of intermediate 1,3,6,8-tetramethyl-9H-carbazole with tris(4-bromo-2,6-dimethylphenyl)borane following the same procedure for synthesis of TBP-3MCz generated the pure TB-3MCz as a pale yellow power (Yield: 71%). ^{1}H NMR (500 MHz, Chloroform-*d*) δ 7.75 (d, J = 1.7 Hz, 6H), 7.18 (s, 6H), 6.93 (d, J = 1.7 Hz, 6H), 2.49 (s, 18H), 2.21 (s, 18H), 2.02 (s, 18H). ^{13}C NMR (126 MHz, $CDCl_3$) δ 146.36, 143.82, 140.81, 139.52, 130.97, 130.15, 128.99, 124.25, 121.27, 117.84, 29.74, 23.10, 21.16, 19.56. HRMS calcd for $C_{72}H_{72}BN_3$ 989.5819 (1012.5717, M+Na), found 1012.5723 [M+Na]. (Supplementary Fig. 37-39)

Excited state lifetimes and rate constants calculations. In the transient PL decay curves of TADF materials, they usually follow multiple exponential decay consisting of prompt fluorescence and delayed fluorescence. Specially, due to the conformation distribution effect, the delayed component of D-A type TADF molecules usually follows multiple exponential decay rather than single exponential decay as that in diluted solution (Supplementary Figure 15c). The transient PL decays were fitted by the following equation:

$$I(t) = A_1 \exp\left(-\frac{t}{\tau_1}\right) + A_2 \exp\left(-\frac{t}{\tau_2}\right) + A_3 \exp\left(-\frac{t}{\tau_3}\right) \quad (1)$$

where τ_1, τ_2 and τ_3 are fluorescence lifetime and the A refers to the integrated arear of each component. And the delayed lifetimes τ_{DF} were averaged from the lifetime of the delayed components:

$$\tau_D = \sum_i A_i \tau_i^2 \,/\, \sum_i A_i \tau_i \quad (2)$$

The exciton density of singlet [S] and triplet [T] rate equation can be expressed as:

$$\frac{d}{dt}\begin{pmatrix}[S_1]\\ [T_1]\end{pmatrix} = \begin{pmatrix}-(k_r^S + k_{nr}^S + k_{RISC}) & k_{RISC}\\ k_{ISC} & -(k_r^T + k_{nr}^T + k_{RISC})\end{pmatrix}\begin{pmatrix}[S_1]\\ [T_1]\end{pmatrix} \quad (3)$$

$$[S_1] = A_1 \exp(-k_p t) + A_2 \exp(-k_d t) \quad (4)$$

$$k_p = 1/\tau_{PF} \quad (5)$$

$$k_d = 1/\tau_{DF} \quad (6)$$

where k_r^S, k_{nr}^S, k_{ISC}, k_r^T, k_{nr}^T and k_{RISC} are the rate constant of radiation transition of singlet state, non-radiation transition of singlet state, ISC, radiation transition of triplet state, non-radiation transition of triplet state and RISC, respectively. The PL quantum yields of prompt fluorescence (Φ_{PF}) and delayed fluorescence (Φ_{DF}) (calculated according to the percentage of prompt fluorescence and delayed fluorescence in the exponential decay fitting) are expressed as:

$$\emptyset_{PF} = \frac{k_r^S}{k_r^S + k_{ISC} + k_{nr}^S} \quad (7)$$

$$\emptyset_{DF} = \sum_{n=1}^{\infty} \emptyset_{PF}(\emptyset_{ISC}\emptyset_{RISC})^n = \frac{\emptyset_{PF}\emptyset_{ISC}}{1-\emptyset_{ISC}} \quad (8)$$

Solving the equations, the rate constant of prompt fluorescence (k_p) and delayed fluorescence (k_d) can be given by:[1]

$$k_p, k_d = \frac{k_r^S + k_{nr}^S + k_{nr}^T + k_{RISC}}{2} \times \left(1 \pm \sqrt{1 - \frac{4(k_r^S + k_{nr}^S + k_{ISC})(k_{nr}^T + k_{RISC}) - 4k_{ISC}k_{RISC}}{(k_r^S + k_{nr}^S + k_{ISC} + k_{nr}^T + k_{RISC})^2}}\right) \quad (9)$$

However, the above equation cannot be solved, and approximations have to be made. For TADF materials with high PLQY, the $k_r^S + k_{ISC} >> k_{nr}^S$ can be expected. Moreover, triplet state generally has negligible triple exciton radiative transition and non-radiative transition, and $k_{RISC} >> k_r^T + k_{nr}^T$ can be assumed. Therefore, the rate constant equations can be simplified as:

$$k_r^S = \frac{\emptyset_{PF}}{\tau_{PF}} \quad (10)$$

$$k_{ISC} = \frac{1-\emptyset_{PF}}{\tau_{PF}} = k_P - k_r^S \quad (11)$$

$$k_{RISC} = \frac{k_P k_D \emptyset_{DF}}{k_{ISC}\emptyset_{PF}} \quad (12)$$

Similarly, under the same assumptions, in reference 2,[2] the equations were simplified to be:

$$k_p k_d \approx k_r^S k_{RISC} \quad (13)$$

$$k_{RISC} \approx \frac{k_p k_d}{k_p - k_{ISC}} \quad (14)$$

Moreover, Kaji *et. al.* propose that, under assumption of k_r^T=0 and $k_{nr}^T \approx 0$, the k_{RISC} can be expressed as:[3]

$$k_{RISC} \approx \frac{k_p + k_d}{2} \pm \sqrt{\left(\frac{k_p + k_d}{2}\right)^2 - k_p k_d \left(1 + \frac{\emptyset_{DF}}{\emptyset_{PF}}\right)} \quad (15)$$

Supplementary Table 1. Comparison of the calculated rate constant of RISC using different models.

Molecules	Φ_{PF} %	Φ_{DF} %	τ_{PF} ns	τ_{DF} µs	k_{RISC} [a)] 10^6 s^{-1}	k_{RISC} [b)] 10^6 s^{-1}	k_{RISC} [c)] 10^6 s^{-1}
TBP-3MCz	20.69	59.31	25.82	1.31	2.76	3.69	3.15
TBP-DMAc	40.88	48.12	23.66	3.85	0.52	1.26	0.57
TBP-3aDMAc	31.42	35.58	14.39	44.50	0.0372	0.11	0.048
TRZ-3MCz	26.53	61.47	20.02	1.63	1.94	2.97	2.10
TB-3MCz	27.63	55.37	17.20	1.94	1.43	2.49	1.58

a) Model from *Nat. Photonics* **2012**, 6, 253-258;[1] b) model from *Nat. Photonics* **2020**, 14, 636-642;[2] c) model from *Nat. Photonics* **2020**, 14, 643–649.[3]

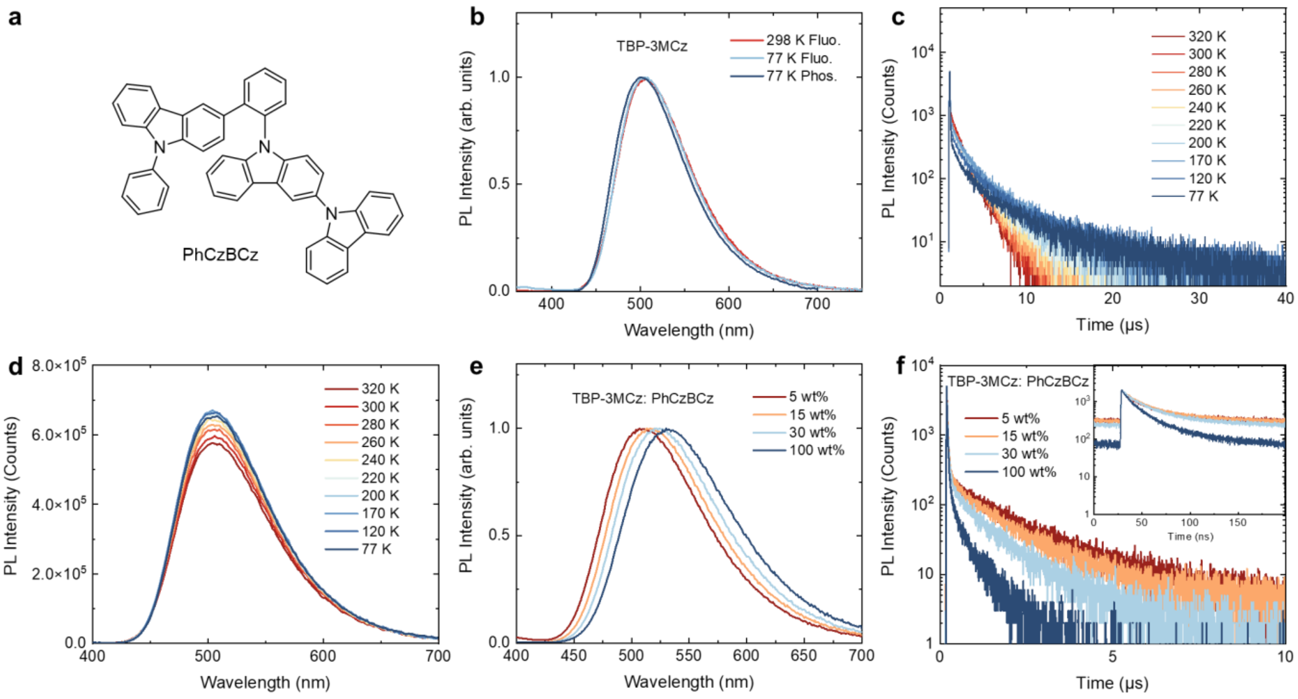

Supplementary Fig. 1 Photo-physical properties of TBP-3MCz. **a** Molecular structure of the PhCzBCz host; **b** Fluorescence spectra measured at 298 K and 77 K and phosphorescence spectrum measured at 77 K (delayed 5 ms) of the 15 wt% TBP-3MCz: PhCzBCz film; Temperature-dependent **c** transient PL decay characters and **d** PL spectra of the 15 wt% TBP-3MCz: PhCzBCz film; **e** PL and **f** transient PL decay characteristics of the TBP-3MCz: PhCzBCz films with different doping concentrations. As the doping concentration increases, the spectrum redshifts while the prompt and delayed fluorescence lifetimes decrease. This is a normal phenomenon in TADF because of the increased polarity and exciton quenching from intermolecular interactions. To prevent intermolecular interactions, we used 15 wt% doping concentration for investigation and device fabrication. Source data are provided as a Source Data file.

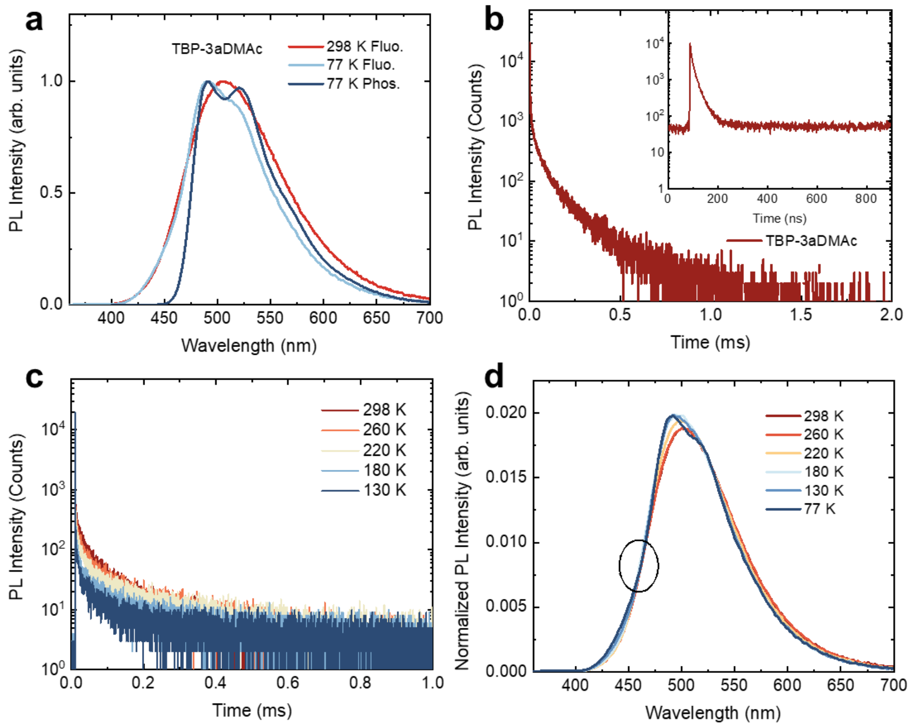

Supplementary Fig. 2 Photo-physical properties of TBP-3aDMAc. **a** Fluorescence spectra measured at 298 K and 77 K and phosphorescence spectrum measured at 77 K (delayed 5 ms) of the 15 wt% TBP-3aDMAc: PhCzBPCz film; **b** Transient PL decays of 15 wt% TBP-3aDMAc: PhCzBCz film measured at different timescales; temperature-dependent **c** transient PL decays and **d** area-normalized PL spectra of 15 wt% TBP-3aDMAc: PhCzBCz film. An iso-emissive point can be found in the area-normalized spectra, indicating the emission from two distinct emitting species. Source data are provided as a Source Data file.

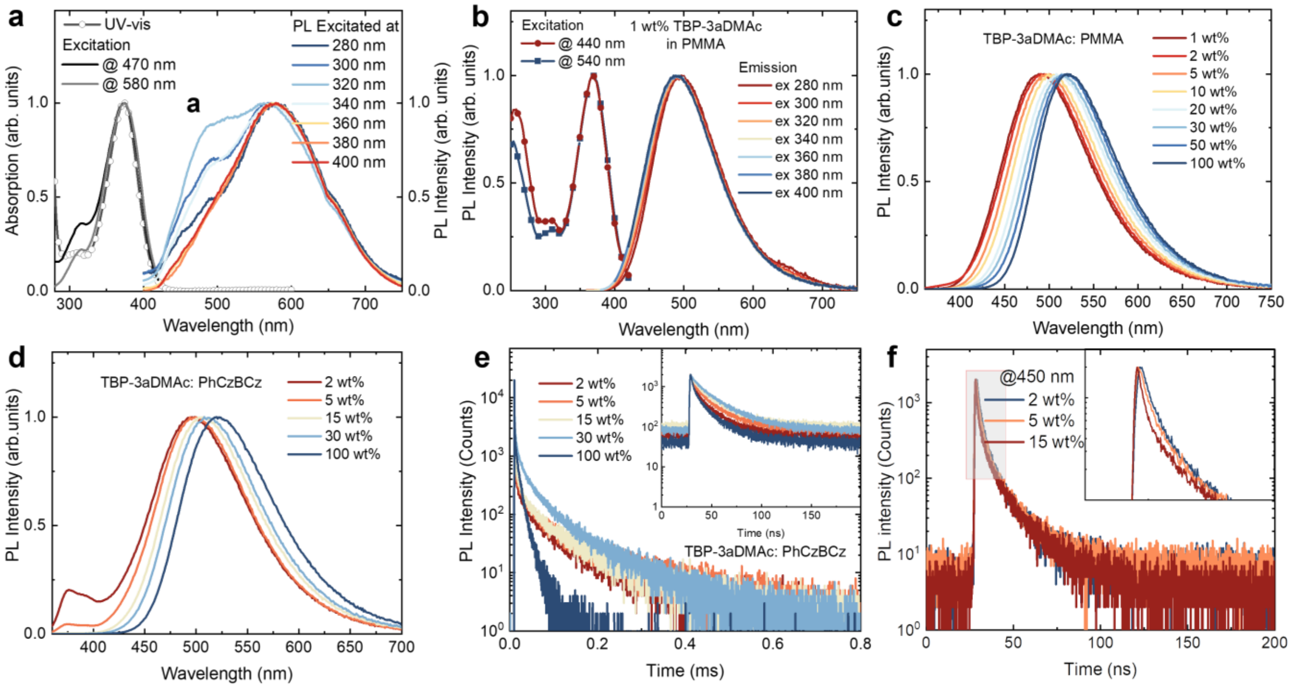

Supplementary Fig. 3 Investigation of the dual conformation characteristics of TBP-3aDMAc. **a** UV-vis absorption, excitation and PL spectra of the TBP-3aDMAc dichloromethane solution (10^{-5} M); **b** excitation and PL spectra of the 1 wt% TBP-3aDMAc: PMMA film; concentration-dependent PL spectra of TBP-3aDMAc in **c** PMMA and **d** PhCzBCz host (excited at 320 nm); **e** Transient PL decays of the TBP-3aDMAc: PhCzBCz film with different doping concentrations (measured in the peak wavelength); **f** transient PL decays of the TBP-3aDMAc: PhCzBCz film with different doping concentrations measured at 450 nm for the quasi-axial emission band. The different conformers have different transition dipole moments, and therefore, in diluted dichloromethane with high polarity, the two-emission band can be separated, which can be more distinctive when changing the excitation wavelengths. In the 15 wt% TBP-3aDMAc: PhCzBCz film, the FRET is complete and the short wavelength emission can only be observed by time-resolved spectra (Supplementary Fig. 11c). Source data are provided as a Source Data file.

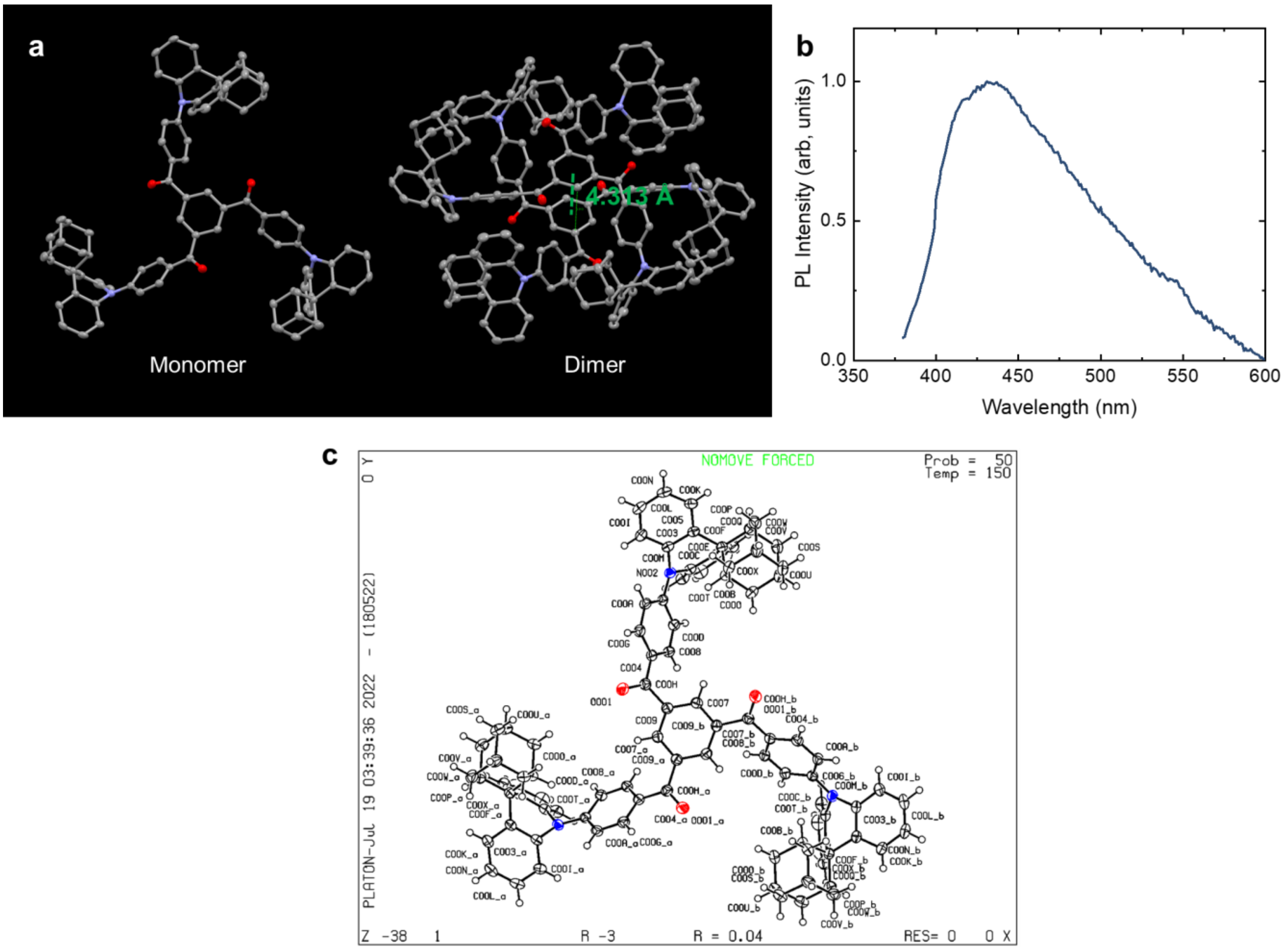

Supplementary Fig. 4 Single crystal analysis of TBP-3aDMAc (CCDC: 2191190). **a** Single crystal structure of TBP-3aDMAc monomer and dimer with quasi-axial conformation, the green line denotes the π-π interaction distance of 4.313 Å; **b** PL spectrum of the TBP-3aDMAc single crystal; **c** Single crystal structure of TBP-3aDMAc, with thermal ellipsoids shown at 50% probability. The X-ray crystallographic data can be obtained from CCDC (10.5517/ccdc.csd.cc2ck3km).

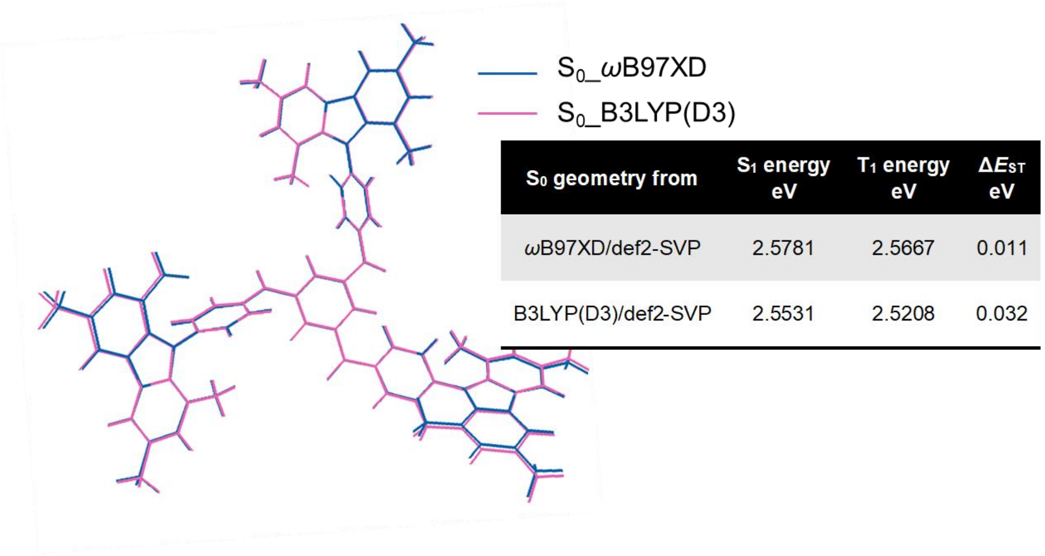

Supplementary Fig. 5 Comparison of the optimized geometries using different methods and the vertical transition energies based on the optimized geometries (in ωB97XD*/def2-SVP level).

Supplementary Table 2. Comparison of the calculated excited state energies of TBP-3MCz calculated by different methods

Methods	ωB97XD*/ def2-SVP	DSD-PBEP86/ def-TZVP [a)]	ωB97XD/ def2-SVP	B3LYP/ def2-SVP	M062x/ def2-SVP	PBE0/ def2-SVP	Exp. [b)]
S_1/ eV	2.553	2.594	3.813	2.428	3.559	2.636	~2.60
T_1/ eV	2.521	-	2.977	2.398	3.104	2.584	~2.55
ΔE_{ST}/ eV	0.032	-	0.836	0.030	0.455	0.052	0.05

a) Considering the computational cost, only def-TZVP basis set can be adopted for the double hybrid functional calculation, and we cannot assess to the lowest triplet state after the double correction of the initial 10 triplet states in the calculation (output energy >8 eV); b) experimental singlet and triplet energy in diluted toluene solution, estimated from the peak wavelength of the fluorescence and phosphorescence spectra measured at 77 K.

Supplementary Table 3. TD-DFT calculation results of the investigated molecules.

	D-A angels	Singlet			Triplet		SOCME	
	°		eV	f		eV	cm^{-1}	
TBP-3MCz	77.42	S_1	2.553	0.0398	T_1	2.521	S_1-T_1	0.077
	78.78	S_2	2.554	0.0393	T_2	2.521	S_1-T_2	0.038
	77.47	S_3	2.558	0.0071	T_3	2.522	S_1-T_3	0.014
TBP-DMAc	80.76	S_1	2.476	0.0002	T_1	2.470	S_1-T_1	0.007
	84.26	S_2	2.476	0.0002	T_2	2.470	S_1-T_2	0.004
	84.2	S_3	2.477	0.0002	T_3	2.471	S_1-T_3	0.002
TBP-3aDMAc (QA)	2.93	S_1	3.359	0.7308	T_1	2.732	S_1-T_1	0.298
	5.64	S_2	3.364	0.7879	T_2	2.749	S_1-T_2	0.481
	2.94	S_3	3.442	0.1294	T_3	2.754	S_1-T_3	0.663
TBP-3aDMAc (2QA+1QE)	79.23	S_1	2.864	0.0002	T_1	2.710	S_1-T_1	0.019
	6.38	S_2	3.273	0.2866	T_2	2.723	S_1-T_2	0.025
	3.87	S_3	3.310	0.2120	T_3	2.854	S_1-T_3	0.113
TBP-3aDMAc (1QA+2QE)	78.77	S_1	2.797	0.0004	T_1	2.671	S_1-T_1	0.017
	77.25	S_2	2.802	0.0002	T_2	2.788	S_1-T_2	0.052
	2.13	S_3	3.148	0.0007	T_3	2.793	S_1-T_3	0.020
TBP-3aDMAc (QE)	75.78	S_1	2.608	0.0007	T_1	2.601	S_1-T_1	0.011
	75.35	S_2	2.608	0.0006	T_2	2.601	S_1-T_2	0.013
	75.80	S_3	2.608	0.0011	T_3	2.601	S_1-T_3	0.006
TRZ-3MCz	83.55	S_1	2.580	0.0164	T_1	2.565	S_1-T_1	0.050
	85.03	S_2	2.582	0.0248	T_2	2.569	S_1-T_2	0.040
	84.09	S_3	2.587	0.0036	T_3	2.569	S_1-T_3	0.022
TB-3MCz	83.99	S_1	2.766	0.0097	T_1	2.751	S_1-T_1	0.050
	84.1	S_2	2.766	0.0097	T_2	2.752	S_1-T_2	0.040
	84.01	S_3	2.771	0.0001	T_3	2.758	S_1-T_3	0.022

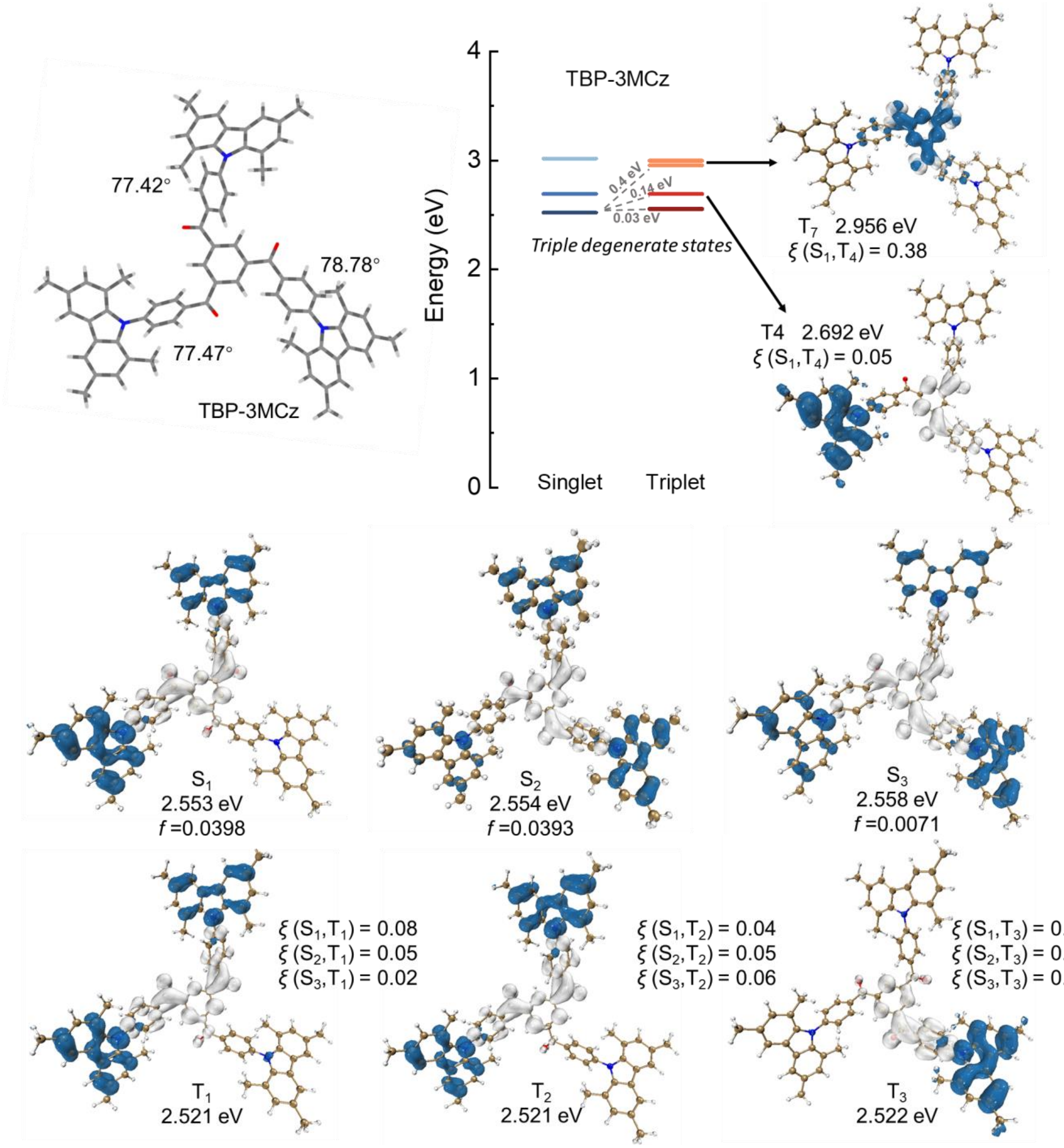

Supplementary Fig. 6 Optimized ground state geometry, excited state energy levels, calculated hole-electron distributions, oscillator strengths (*f*), and spin-orbit coupling matrix elements (H_{SOC}, ξ) of TBP-3MCz. The n-π* transition triplet state of the TBP moiety (T_7) with large SOC is 0.4 eV higher than the singlet state of TBP-3MCz, which indicates that it would have little interactions with the S_1 state in the spin-flipping process. And the energetically closer T_4 state is a long-range CT state with small SOC.

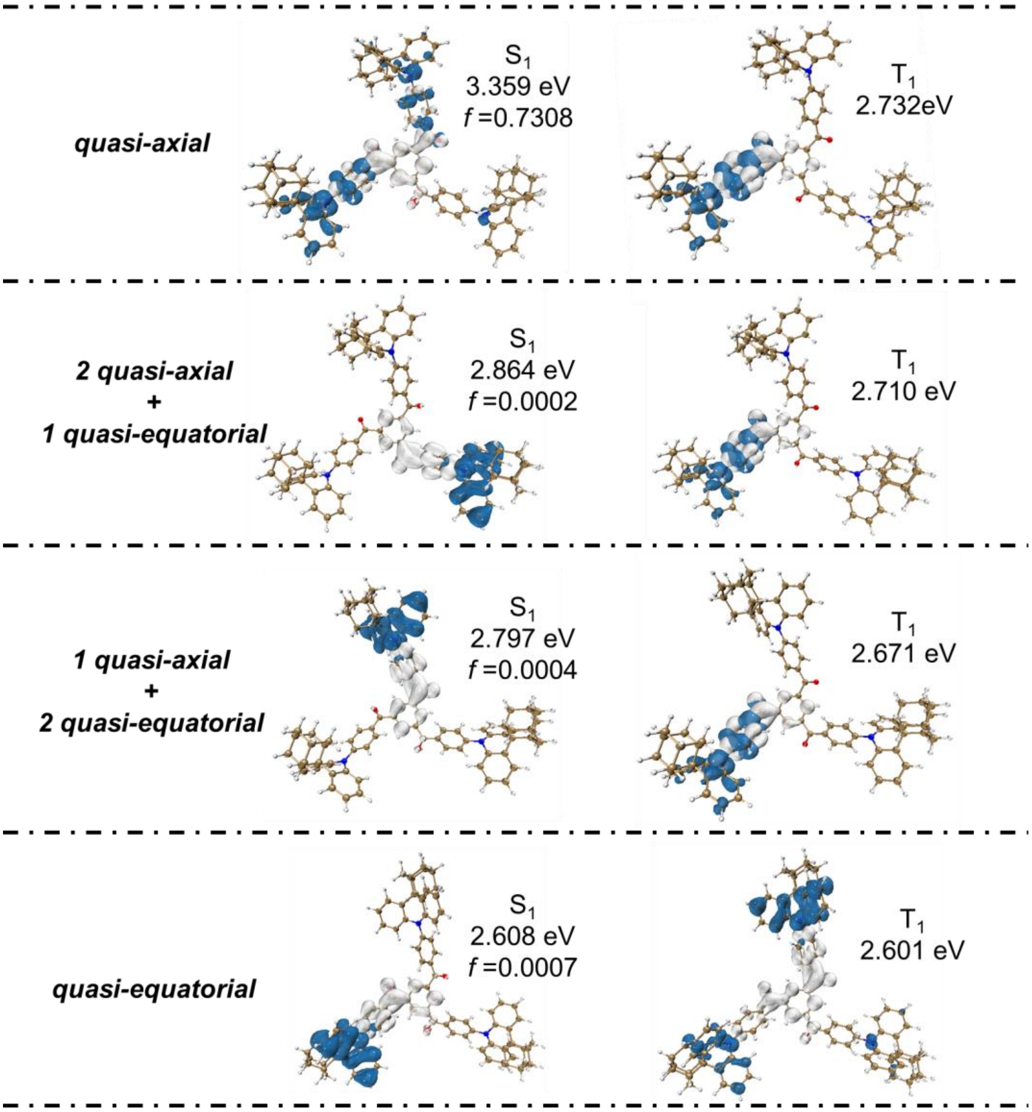

Supplementary Fig 7. Optimized geometries of TBP-3aDMAc and the hole-electron distributions of singlet and triplet states.

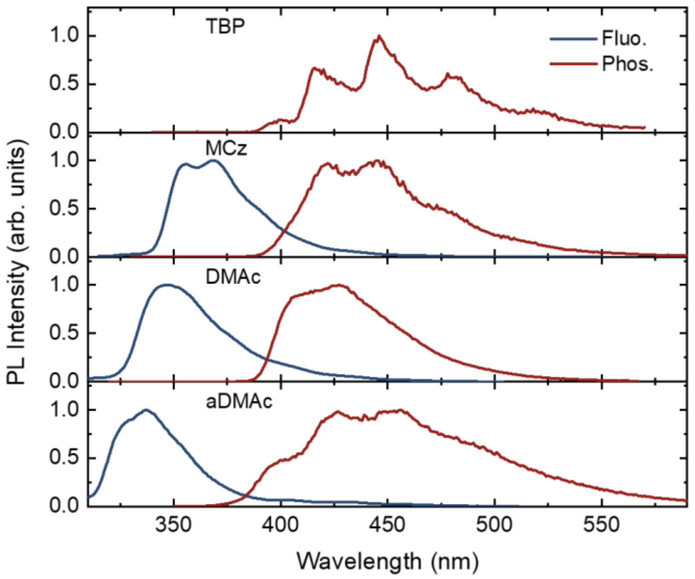

Supplementary Fig. 8 Fluorescence and phosphorescence spectra of the donor and acceptor fragments of the investigated molecules in diluted toluene solution (10^{-4} M). Source data are provided as a Source Data file.

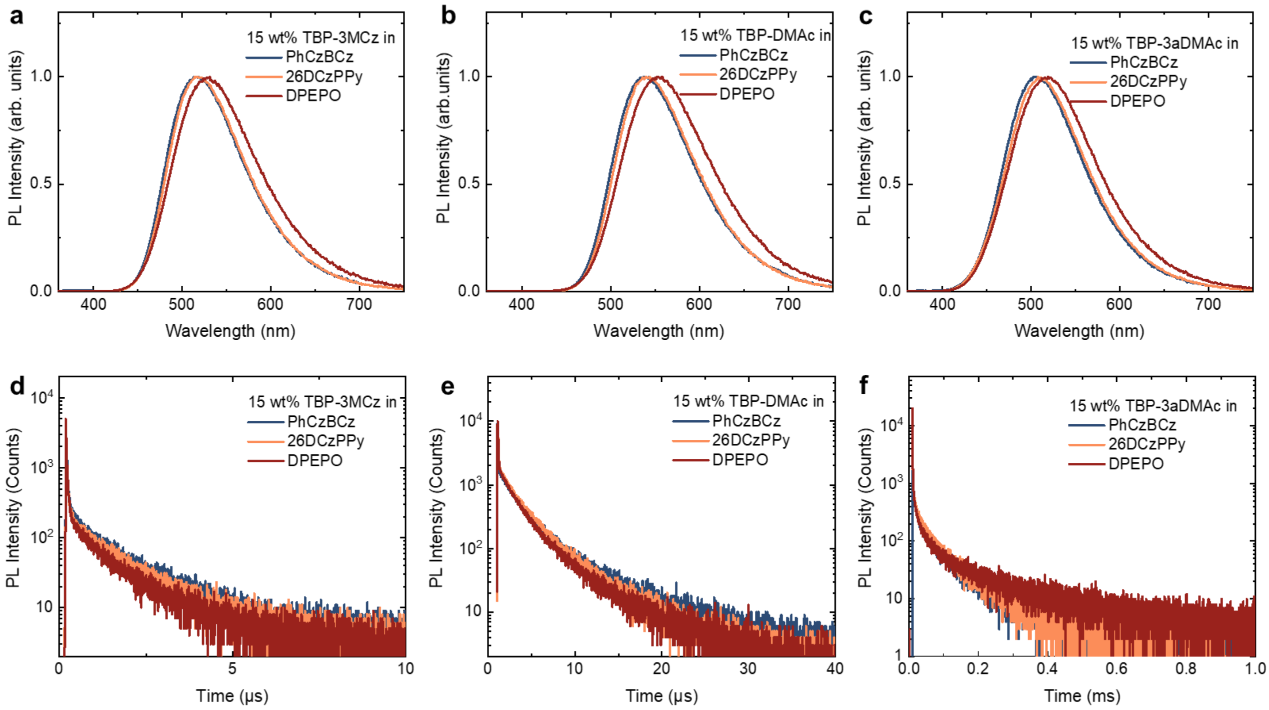

Supplementary Fig 9. Photophysical properties of the emitters in different host. **a-c** PL and **d-f** transient PL decay characteristics of 15 wt% TBP-3MCz, TBP-DMAc and TBP-3aDMAc in hosts with different polarities. 26DCzPPy: 2,6-bis[3-(9H-Carbazol-9-yl)phenyl]pyridine; DPEPO: Bis[2-(diphenylphosphino)phenyl]ether oxide. Source data are provided as a Source Data file.

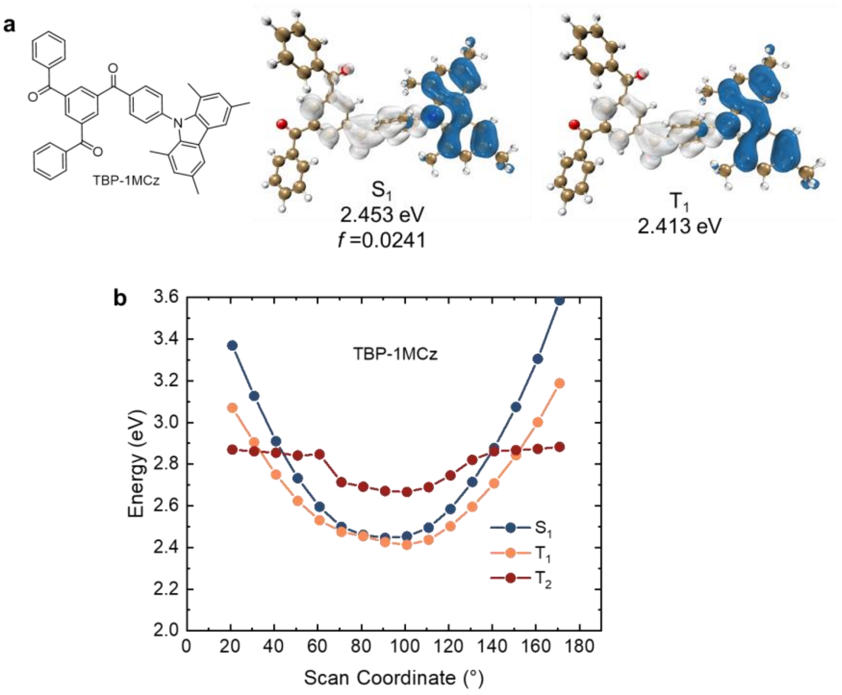

Supplementary Fig. 10 TD-DFT calculation of TBP-1MCz. **a** Molecular structure and hole-electron distributions of singlet and triplet state of TBP-1MCz and **b** the PES of the S_1, T_1 and T_2 of TBP-1MCz states, calculated in B3LYP/6-31G* level. According to the S_1, T_1 and T_2 energies, the thermally accessible conformers of TBP-1MCz (50°-130°) have a large energy gap (>0.2 eV) between S_1 and T_2, indicating that the influence of the higher energy triplet state is less significant as the changing of the conformation. Source data are provided as a Source Data file.

Supplementary Table 4. TD-DFT calculated ΔE_{ST} in the ground state geometries (QA or QE conformer) and average ΔE_{ST} according to Boltzmann distribution on the scanned S_1 and T_1 PES in Fig 2.

	ΔE_{ST} in S_0 geometry meV	S_1 average ΔE_{ST} [a)] meV	T_1 average ΔE_{ST} [a)] meV
TBP-1MCz	40.4	23.6	25.6
TBP-1DMAc	462 (QA 33%)/ 7.7 (QE 67%) [b)]	25.4	75.4
TBP-1aDMAc	463 (QA 94%)/ 8.5 (QE 6%) [b)]	28.7	153.8 (QA 11%/ QE 89%)

a) The conformer distributions were estimated by Boltzmann distribution: $\%Conformer\ i = \frac{exp\ (-\frac{E_i}{k_b T})}{\sum_j exp\ (-\frac{E_j}{k_b T})}$, where E_i is the conformational energy of conformer i calculated by DFT or TD-DFT, k_b is Boltzmann constant and T is the ambient temperature (298 K); and the average energies were calculated by timing TD-DFT calculated ΔE_{ST} in each geometry with the *%Conformer i* of the corresponding scanned singlet or triplet PES.

b) The ΔE_{ST} calculated in quasi-axial (QA) and quasi-equatorial (QE) conformation and the percentages of each conformation calculated according to the geometry energies and Boltzmann distributions.

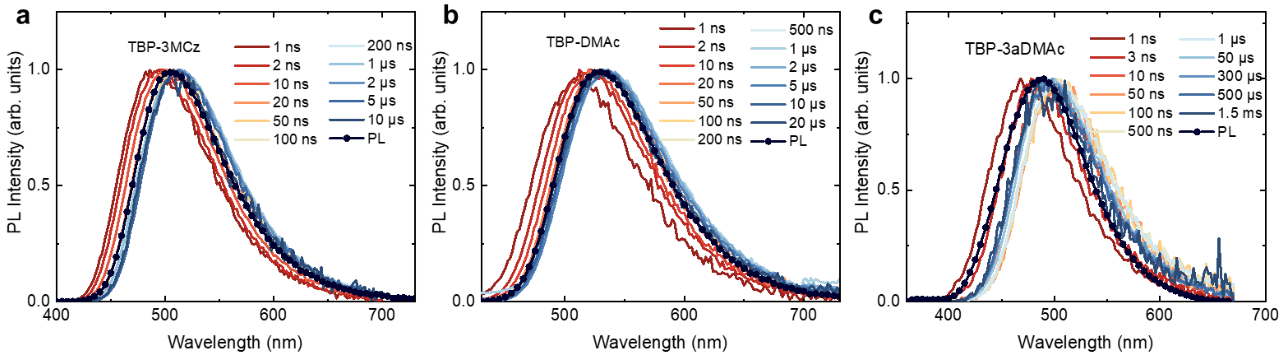

Supplementary Fig. 11 Time-resolved PL spectra and steady-state PL spectra of the 15 wt% **a** TBP-3MCz, **b** TBP-DMAc and **c** TBP-3aDMAc doped PhCzBCz films. Source data are provided as a Source Data file. Source data are provided as a Source Data file.

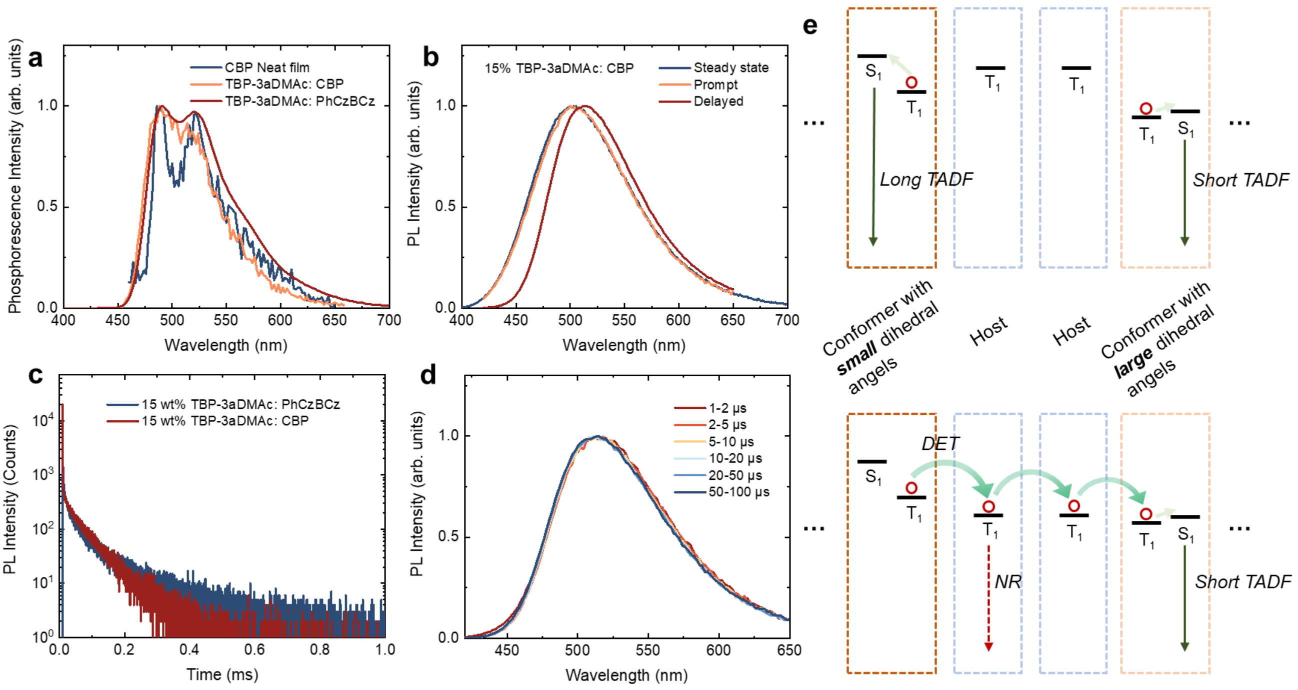

Supplementary Fig. 12 Investigation of the triplet state distribution and exciton diffusion in the host-guest system. **a** Phosphorescence spectra of CBP neat film, 15 wt% TBP-3aDMAc: CBP film and 15 wt% TBP-3aDMAc: PhCzBCz film; **b** Comparison of the steady state PL spectra, prompt fluorescence spectra (0-500 ns) and delayed fluorescence spectra (> 1 μs) of the 15 wt% TBP-3aDMAc: CBP film; **c** comparison of the transient PL decay of the 15 wt% TBP-3aDMAc doped CBP and PhCzBCz films; **d** time-resolved PL spectra of the delayed components of the 15 wt% TBP-3aDMAc: CBP film; **e** schematic illustration of the TADF emission of TBP-3aDMAc with different conformers in host with high or low triplet energy (DET: Dexter energy transfer, NR: nonradiative transition). Source data are provided as a Source Data file.

Supplementary Note

Delayed lifetime distributions in host-guest systems. The transient lifetime decay curve also contains the information of conformation distribution.[4] A settled conformer has settled emission transient decay lifetime, showing double exponential decay with one prompt fluorescence component and delayed fluorescence component:

$$I(t) = A_{PF}e^{-\frac{t}{\tau_{PF}}} + A_{DF}e^{-\frac{t}{\tau_{DF}}} \quad (16)$$

In the amorphous film state, the broad conformation distribution would lead to multiple exponential decay character, as the different transient decay character of the sample in diluted degassed toluene solution (Supplementary Fig. 15c) and amorphous film state (Figure 5c). The transient PL decay of the delayed fluorescence in amorphous film state is the integration of the multiple single exponential decay from different conformers with different lifetime, and can be represented as:

$$I_{DF}(t) = \sum_i A_i e^{-\frac{t}{\tau_i}} \quad (17)$$

If we use multiple specific τ_j values (covering the whole lifetime region) to fit the transient decay curve, the obtained amplitude values (A_j) from the fitting result can represent the contribution of the component with the given lifetime. The percentage of each component with the given lifetime (p_j) can be calculated as:

$$p_j = A_j\tau_j / \sum_i A_i\tau_i \quad (18)$$

Plotting the p_j against lifetime, the distribution of delayed lifetime can be obtained (Supplementary Fig. S13).

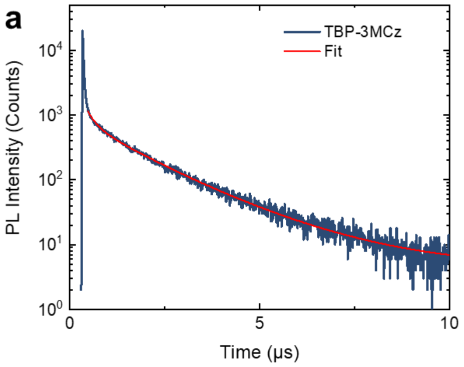

j	τ_j (ns)	A_j (Fitted)
1	200	40.50
2	500	43.55
3	1000	336.18
4	1500	108.97
5	2000	37.63
6	2500	33.14
7	3000	28.79
8	3500	17.53
9	4000	10.80
10	4500	0.01

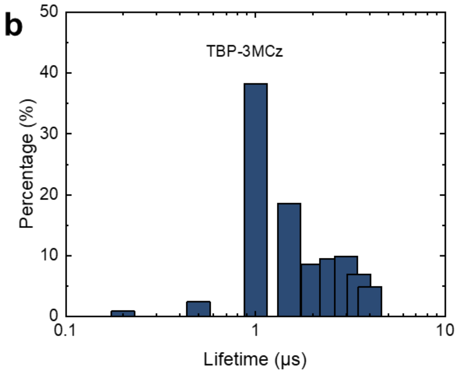

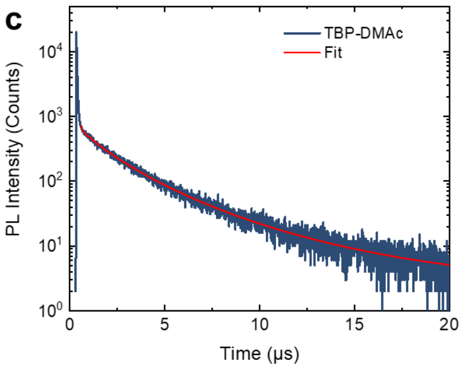

j	τ_j (ns)	A_j (Fitted)
1	200	0.00
2	500	46.77
3	1000	81.00
4	1500	116.65
5	2000	115.47
6	2500	100.33
7	3000	66.88
8	3500	34.15
9	4000	14.15
10	4500	5.98

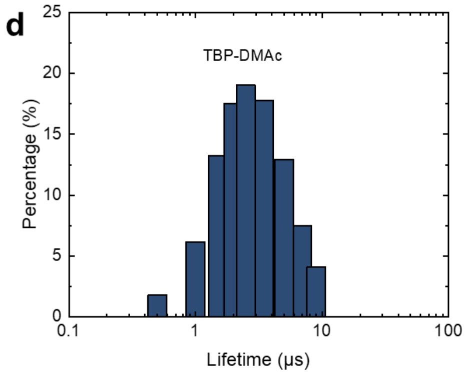

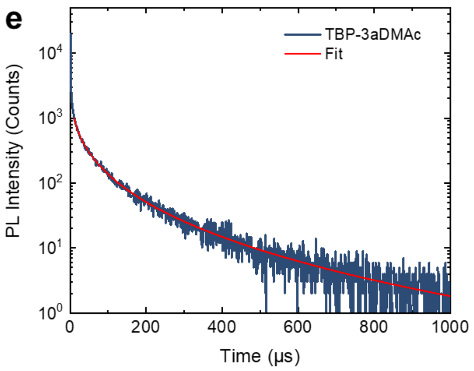

j	τ_j (ns)	A_j (Fitted)
1	2000	0.00
2	5000	197.87
3	10000	227.44
4	20000	160.07
5	30000	205.45
6	50000	171.47
7	80000	92.29
8	120000	46.84
9	150000	32.91
10	200000	21.97
11	300000	7.06
12	500000	2.90
13	800000	1.00

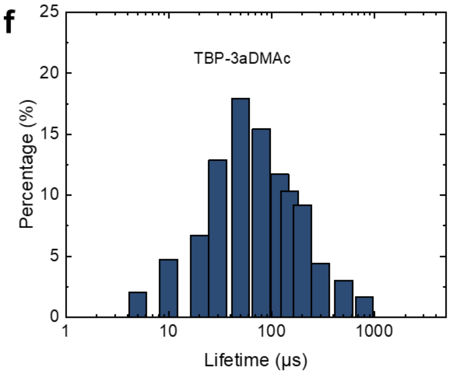

Supplementary Fig. 13 Investigation of the delayed lifetime distribution. Transient PL decay characters of the **a** 15 wt% TBP-3MCz: PhCzBCz, **c** 15 wt% TBP-DMAc: PhCzBCz and **e** 15 wt% TBP-3aDMAc: PhCzBCz films. Delayed lifetime distribution of the **b** 15 wt% TBP-3MCz, **d** TBP-DMAc and **f** TBP-3aDMAc doped PhCzBCz films. The τ_j are given parameters in multiple exponential decay fitting and A_j are fitted parameters. As can be seen in the delayed lifetime distribution, the 15 wt% TBP-3MCz: PhCzBCz system with the shortest excited state lifetime also shows a narrow lifetime distribution, while the 15 wt% TBP-3aDMAc: PhCzBCz has a broad delayed lifetime distribution. The conformation distribution, especially the dihedral angel distribution in the amorphous film state, differentiates the RISC process in different molecules, causing a broad distribution of delayed fluorescence lifetime. TBP-3MCz with confined conformation distributions results in narrow and short delayed lifetime distributions. Source data are provided as a Source Data file.

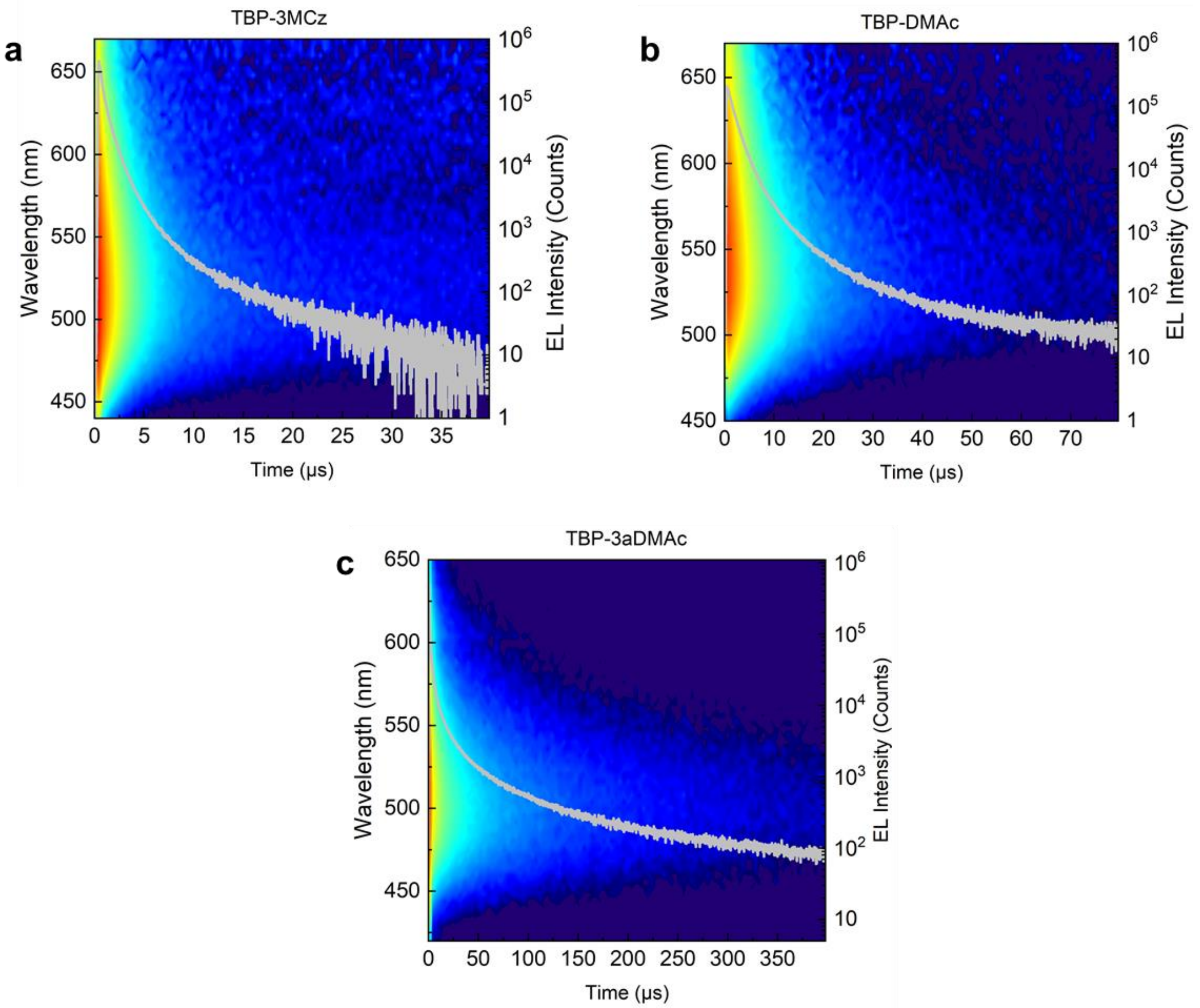

Supplementary Fig. 14 Time-resolved EL measurement of the OLED device. Time-resolved EL spectra of the OLED devices based on **a** 15 wt% TBP-3MCz, **b** 15 wt% TBP-DMAc and **c** 15 wt% TBP-3aDMAc doped PhCzBCz films, and the grey lines show the integrated intensities as a function of delayed times. The device structures for measurements are ITO/ PEDOT: PSS (30 nm)/ 15 wt% TADF emitter: PhCzBCz (30 nm)/ TmPyPb (40 nm)/ CsF (1 nm)/ Al. In the measurement, the devices were driven by a 6 V pulse voltage with a duration of 300 ns, and a -2 V reverse bias was applied after the excitation to eliminate the emission from slow carrier recombination. Source data are provided as a Source Data file.

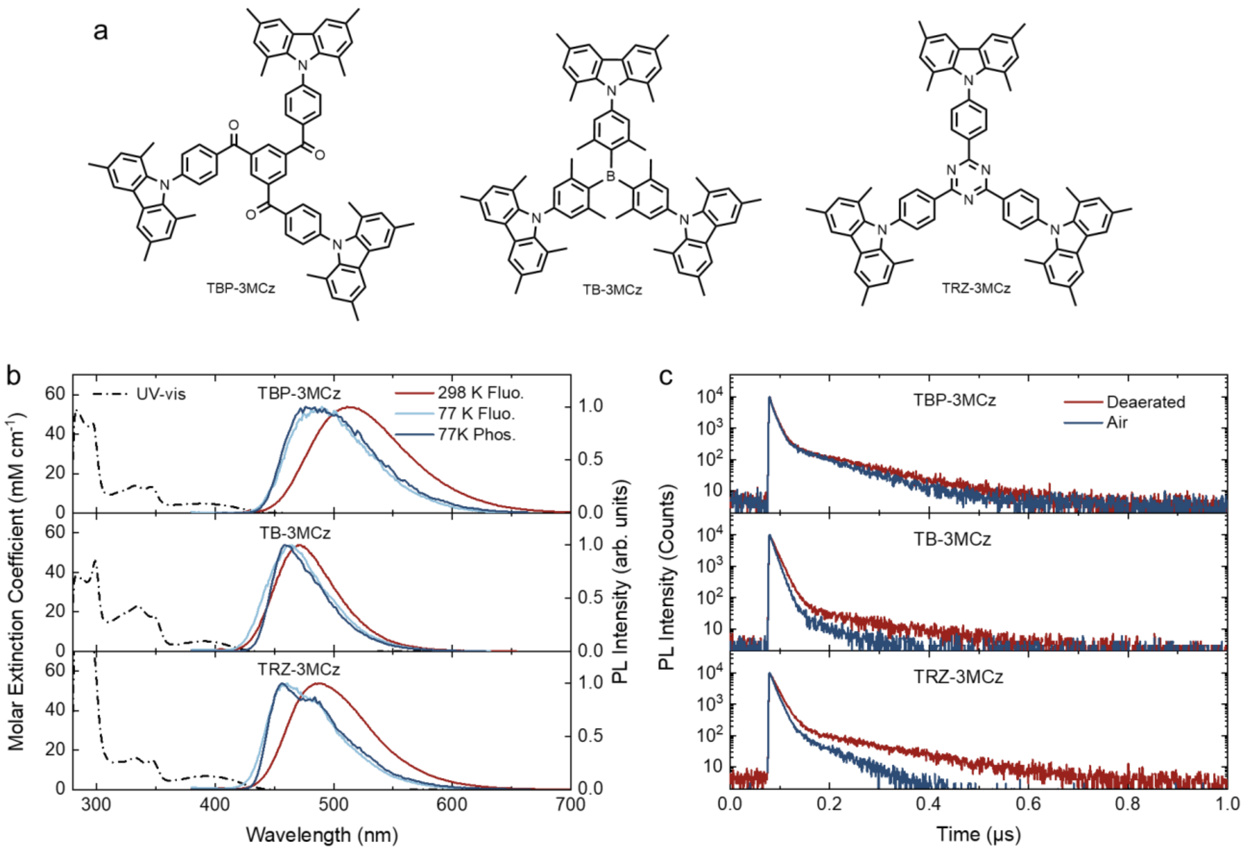

Supplementary Fig. 15 Photo-physical properties of TBP-3MCz, TB-3MCz and TRZ-3MCz in dilute toluene solution (10^{-5} M). **a** Chemical structure of the TADF emitters with MCz donor; **b** UV-vis absorption, fluorescence spectra measured at 298 K and 77 K and phosphorescence spectra measured at 77 K (delayed 5 ms); **c** Transient PL decay characters of the diluted toluene solutions before and after argon bubbling for 10 minutes. Source data are provided as a Source Data file.

Supplementary Table 5. Photo-physical parameters of TBP-3MCz, TB-3MCz and TRZ-3MCz in toluene.

	λ_{em}	Φ_{PL} a)	Φ_{PF}	Φ_{DF}	τ_{PF}	τ_{DF}	k_r^S	k_{ISC}	k_{RISC}
	nm	%	%	%	ns	μs	10^7 s^{-1}	10^7 s^{-1}	10^6 s^{-1}
TBP-3MCz	514	60	34.59	17.31	10.08	0.11	3.43	3.31	13.25
TB-3MCz	471	70	65.56	4.74	12.08	0.14	5.43	2.85	7.88
TRZ-3MCz	487	86	69.61	16.24	13.62	0.14	5.11	2.23	8.89

a) Measured PLQY after argon bubbling for 10 min.

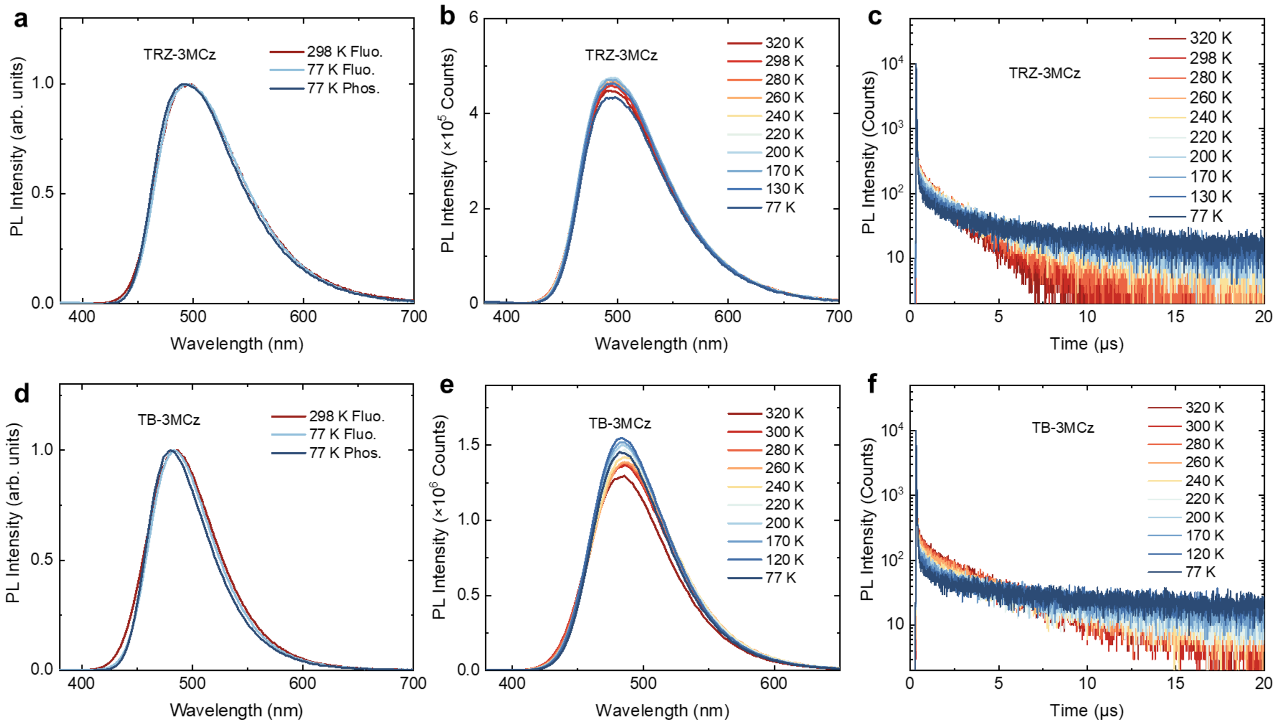

Supplementary Fig. 16 Photo-physical properties of 15 wt% TRZ-3MCz and TB-3MCz doped in PhCzBCz host. **a**, **d** Fluorescence spectra measured at 298 K and 77 K and phosphorescence spectrum measured at 77 K (delayed 5 ms). Temperature-dependent **b**, **e** transient PL decay characters and **c**, **f** PL spectra of the TRZ-3MCz and TB-3MCz doped films respectively. Source data are provided as a Source Data file.

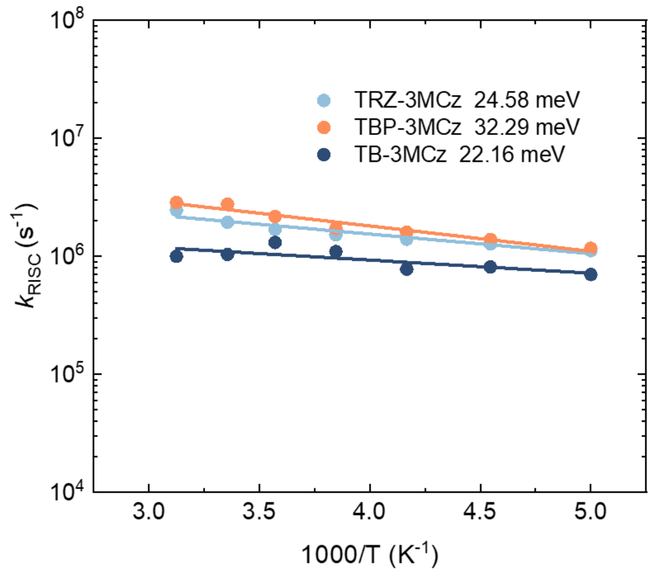

Supplementary Fig. 17 Arrhenius plot of the k_{RISC} of 15 wt% TRZ-3MCz, TBP-3MCz and TB-3MCz doped PhCzBCz films as a function of temperature (1000/T), and the activation energies were fitted by the Arrhenius relationship: $k_{RISC} = A\exp(-E_a/k_BT)$. Source data are provided as a Source Data file.

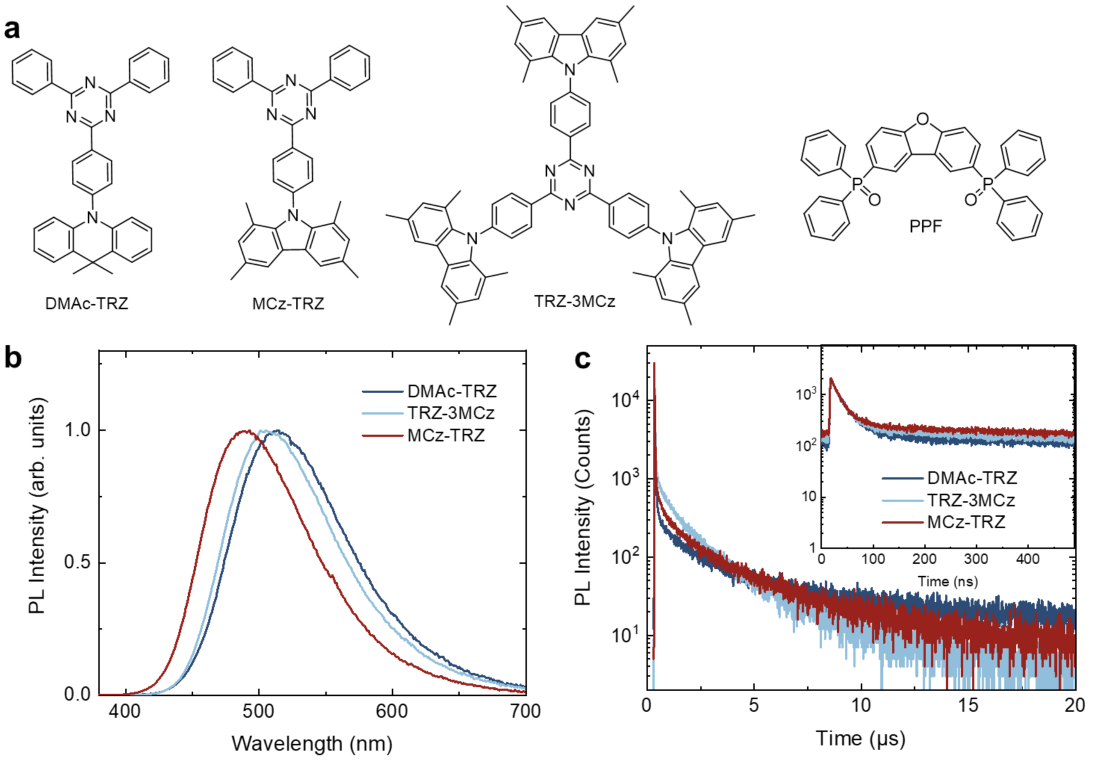

Supplementary Fig. 18 Comparison of the TADF properties of different molecules. **a** Molecular structures, **b** PL spectra and **c** transient PL decay characters of the 10 wt% investigated TADF emitters with a triazine acceptor in PPF host. Source data are provided as a Source Data file.

Supplementary Table 6. Photo-physical parameters of the 10 wt% DMAc-TRZ, MCz-TRZ and TRZ-3MCz in PPF host.

	λ_{em}	Φ_{PL}	Φ_{PF}	Φ_{DF}	τ_{PF}	τ_{DF}	k_r^S	k_{ISC}	k_{RISC}
	nm	%	%	%	ns	μs	10^7 s^{-1}	10^7 s^{-1}	10^6 s^{-1}
DMAc-TRZ	515	83	42.24	40.76	23.64	2.81	1.79	2.08	0.70
TRZ-3MCz	506	87	29.03	57.97	21.19	1.68	1.37	3.14	1.78
MCz-TRZ	488	85	29.83	55.17	17.45	2.37	1.70	5.73	1.20

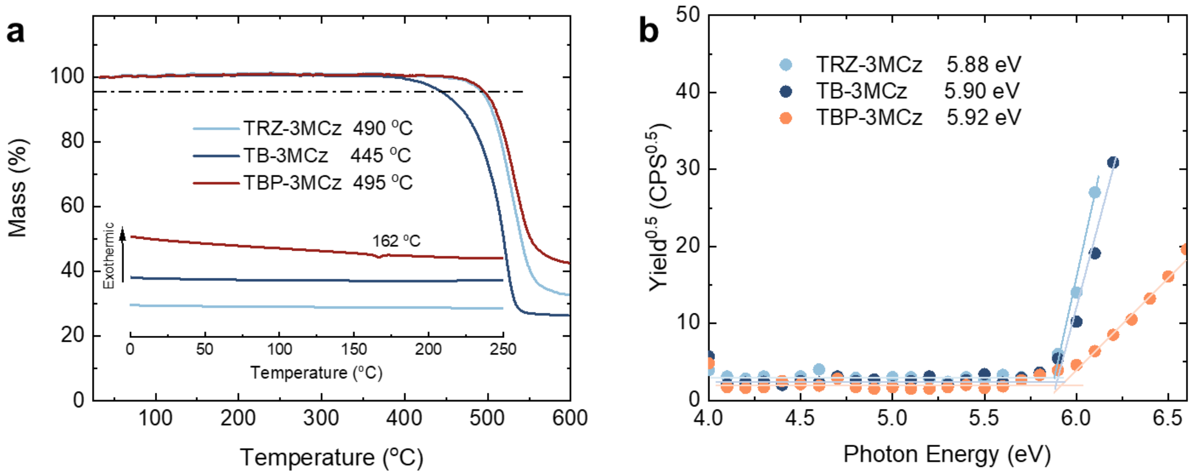

Supplementary Fig. 19 Physical properties. **a** Thermal properties of TRZ-3MCz, TB-3MCz and TBP-3MCz, the dash line shows the thermal deposition temperatures, corresponding to the mass loss of 5%. **b** Photoelectron yield spectra (AC-3) of TRZ-3MCz, TB-3MCz and TBP-3MCz neat films, where the corresponding ionization potentials (IP) were defined by the intersection point of two fitting lines. Source data are provided as a Source Data file.

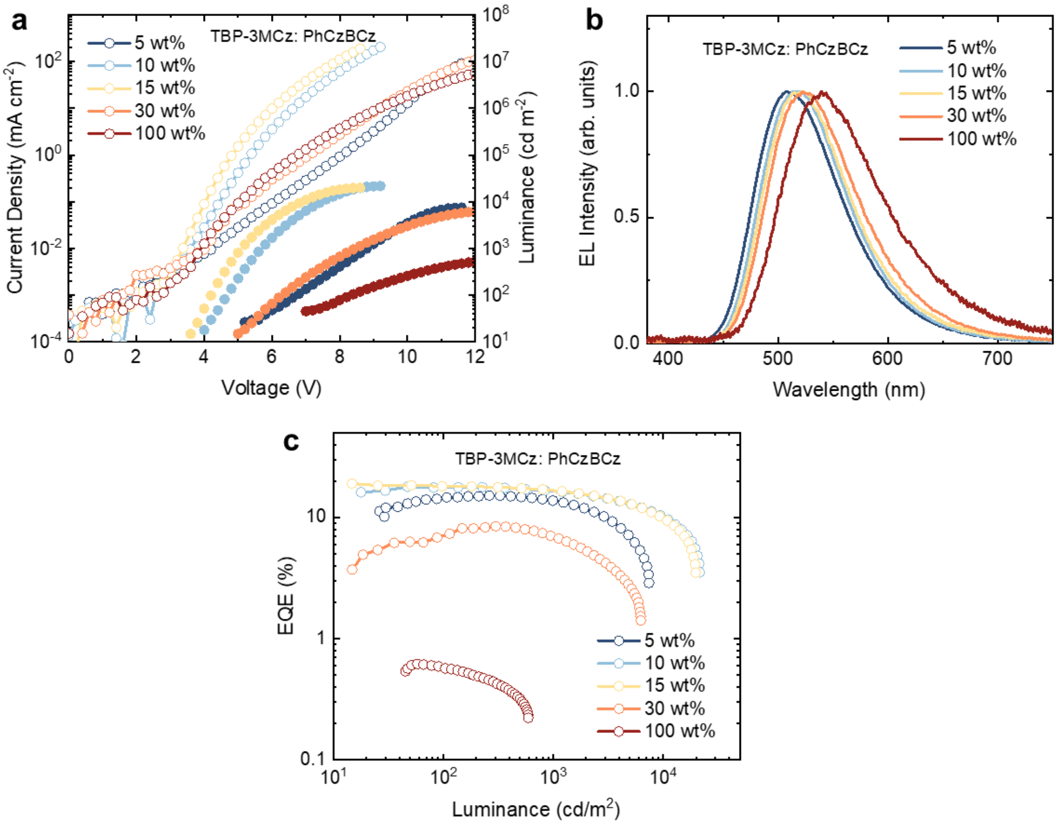

Supplementary Fig. 20 Solution-processed OLED device performance based on TBP-3MCz with different doping concentrations. **a** Current density-voltage-luminance (*J*-*V*-*L*) curves; **b** EL spectra of the devices driven at the current density of 1 mA cm^{-2}; **c** EQE-*L* curves. Source data are provided as a Source Data file.

We had conducted device optimization by varying doping concentration and found that the 15% doping concentration in PhCzBCz host can achieve the highest EQE and low efficiency roll-off. The 5 wt% device having lower efficiency and luminance can be attributed to insufficient energy transfer while further increasing the doping concentration, it would suffer from poor carrier transportation and exciton quenching. The other emitters also show the best performance at 15 wt% doping concentrations. Moreover, at high doping concentration, the involvement of Dexter energy transfer and exciton quenching would increase the complexity for our investigation on conformation distributions. Therefore, we chose 15 wt% concentration for investigations.

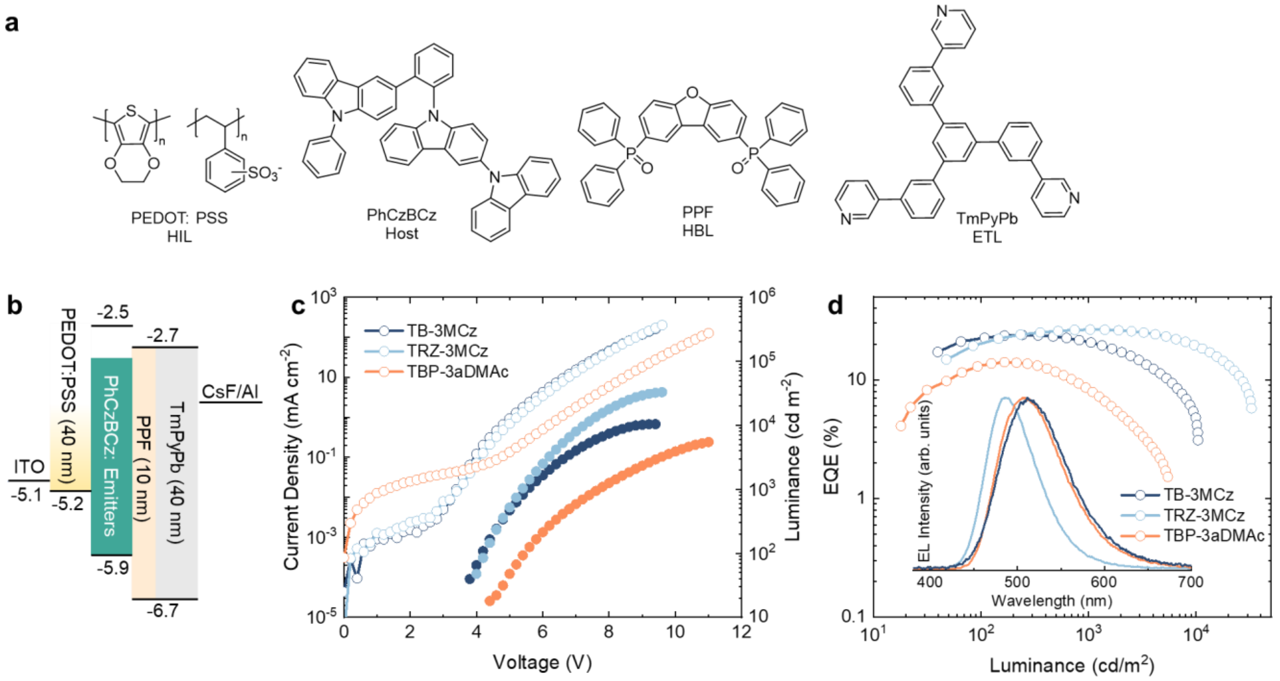

Supplementary Fig. 21 OLED performance of the devices incorporating PPF as blocking layer. **a** Chemical structure of the materials in OLED devices; **b** Device architecture and energy levels of the materials; **c** *J*-*V*-*L* and **d** EQE-*L* curves (insert: EL spectra of the devices driven at the current density of 1 mA cm^{-2}) of the TADF OLEDs. Source data are provided as a Source Data file.

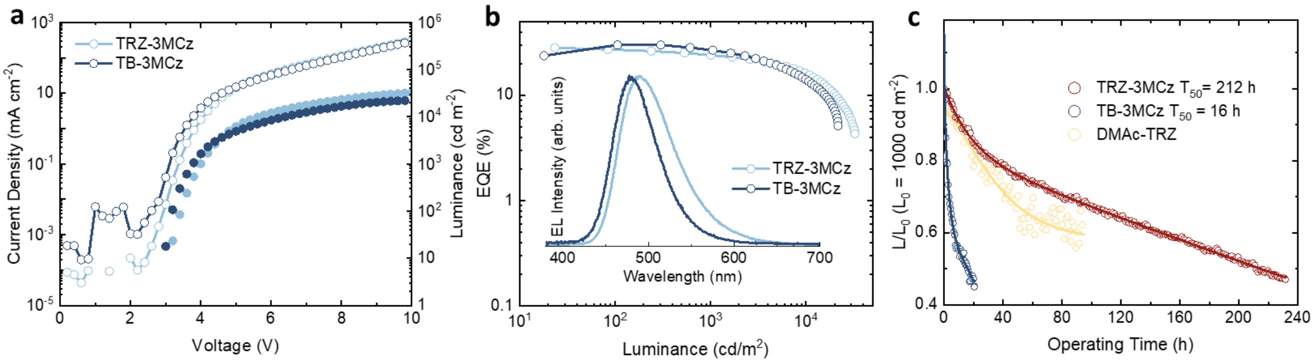

Supplementary Fig. 22 Characterization of the vacuum-evaporated OLED devices. **a** *J*-*V*-*L* curves and **b** EQE-*L* curves (insert: EL spectra of the devices driven at the current density of 1 mA cm^{-2}) of the vacuum-evaporated TADF OLEDs. **c** Luminance of the OLED devices as a function of operation time at initial luminance of around 1000 cd m^{-2}. The device structures are ITO/ HATCN (5 nm)/ TAPC (30 nm)/ TCTA (10 nm)/ PhCzBCz (10 nm)/ 15 wt% TADF emitter: PhCzBCz: (30 nm)/ PPF (10)/ TmPyPb (40 nm)/ LiF (1 nm)/ Al. The operation lifetime of DMAc-TRZ based on the same device structure was also measured for comparison. Source data are provided as a Source Data file.

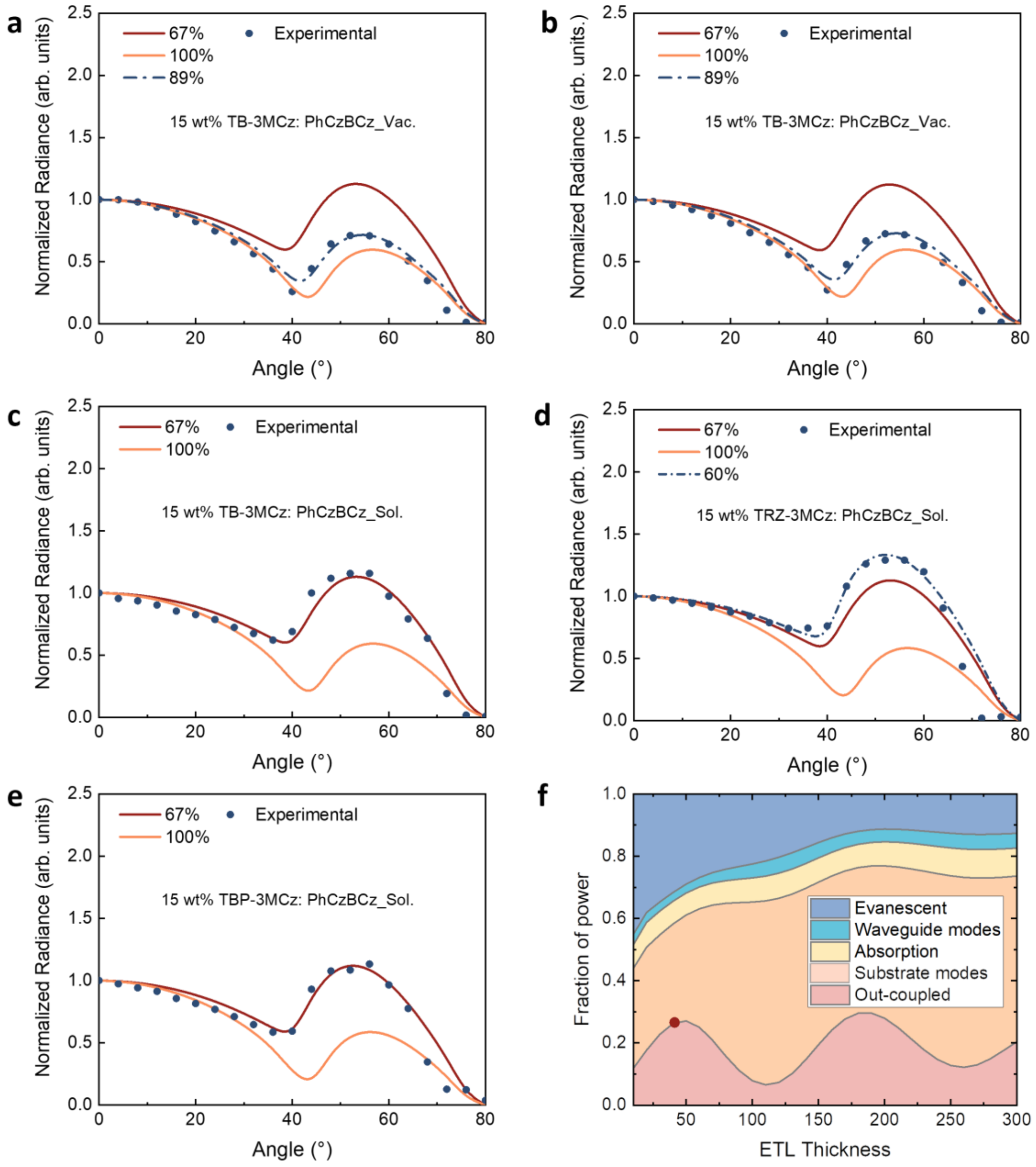

Supplemental Fig. 23 Evaluation of the horizontal orientations of the transition dipole moments in solution-processed and vacuum-evaporated emission layers. Angular-dependent of *p*-polarized PL intensities of the vacuum-evaporated **a** 15 wt% TRZ-3MCz: PhCzBCz and **b** 15 wt% TB-3MCz: PhCzBCz films and solution-processed **c** 15 wt% TRZ-3MCz: PhCzBCz, **d** 15 wt% TB-3MCz: PhCzBCz and **e** 15 wt% TBP-3MCz: PhCzBCz films and the fitted horizontal ratios of the transition dipole moments. **f** Simulated power dissipation to the different optical modes in the solution-processed OLED (stack structure: ITO/ PEDOT: PSS (40 nm)/ 15 wt% TRZ-3MCz: PhCzBCz (30 nm)/ TmPyPb /CsF /Al). Source data are provided as a Source Data file.

The angular-dependence of *p*-polarized light intensity of the solution-processed films were measured and the transition dipole orientations were simulated. Different from the vacuum-evaporated film, the spin-coated film shows no horizontal transition dipole orientation. The 15 wt% TB-3MCz: PhCzBCz film and 15 wt% TBP-3MCz: PhCzBCz film have a horizontal transition dipole orientation ratio of

67%, indicating an isotropic character, while the 15 wt% TRZ-3MCz: PhCzBCz film has slightly vertical orientation. This indicates that the solution-processed OLEDs have an ordinary light-outcoupling. Moreover, we also conducted optical simulation on the 15 wt% TBP-3MCz: PhCzBCz based solution-processed OLED device and the out-coupled fraction of 26.2% was obtained. Therefore, the obtained maximum EQE of 24.4% for TRZ-3MCz based device is reasonable considering the PLQY. It is interesting to note that in the solution-processed OLED with no molecular orientation, the fraction of evanescent field is large, which is originated from the surface plasmon resonance. This loss can be reduced by horizontal orientation of the transition dipole moments.[5] Therefore, improving the molecular orientation in the solution-processed film is an important topic for future development.

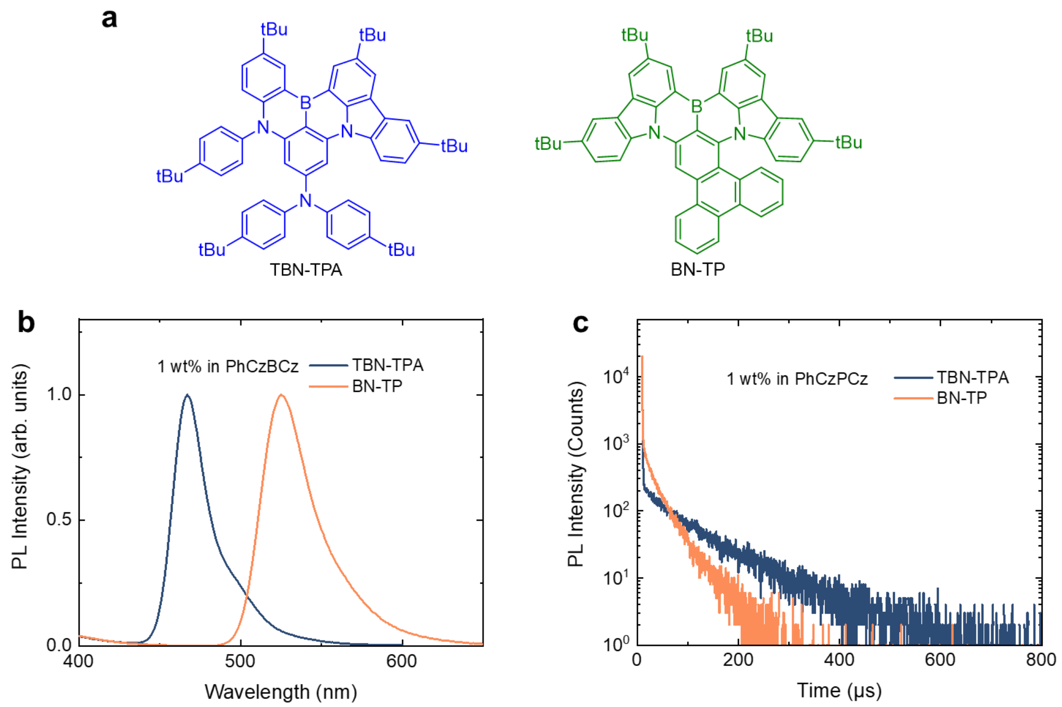

Supplementary Fig. 24 Photo-physical properties of the MR-TADF guests. **a** Molecular structures, **b** PL spectra and **c** transient PL decay characters of the 1 wt% MR-TADF guest in PhCzBCz host. Source data are provided as a Source Data file.

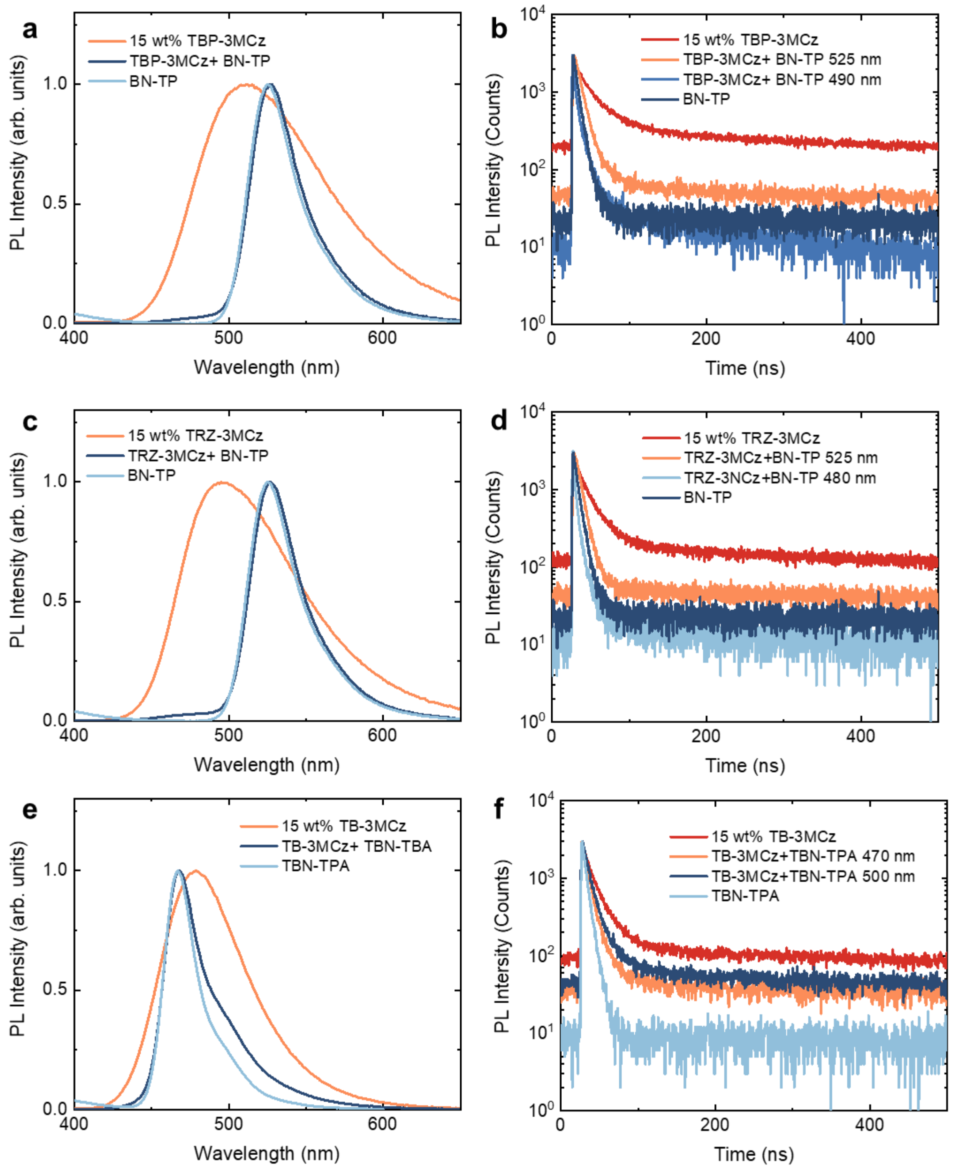

Supplementary Fig. 25 Characterization of the energy transfer between TBP-3MCz, TRZ-3MCz and TB-3MCz TADF assistant hosts and MR-TADF guests. **a**, **c**, **e** PL spectra and **b**, **d**, **f** transient PL decay characters of the doped films with TADF emitters, MR-TADF emitters and TADF+MR-TADF emitters. The significantly shortened fluorescence decay of the TADF assistant host indicates efficient FRET from the TADF assistant host to MR-TADF emitter. Source data are provided as a Source Data file.

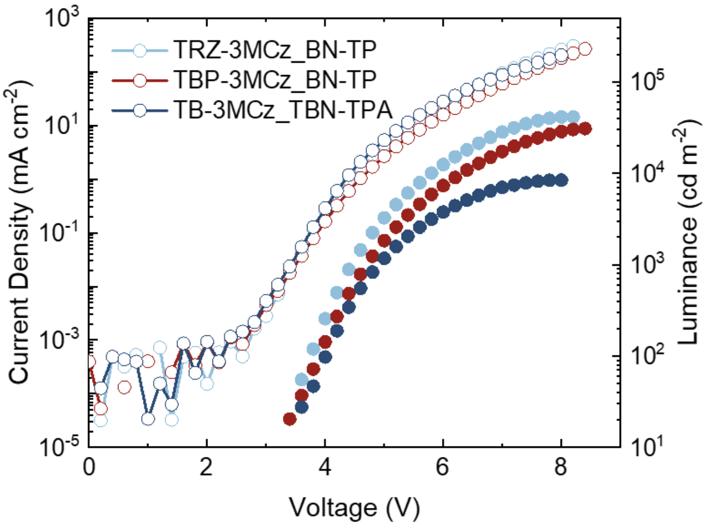

Supplementary Fig. 26. *J-V-L* curves of the sensitized OLED devices. Source data are provided as a Source Data file.

It is interesting to find that, after the doping of MR-TADF, the efficiency roll-off increases slightly in the TRZ-3MCz-based OLED while that based on TBP-3MCz shows reduced efficiency roll-off compared with the OLED without MR-TADF doping. The former can be attributed to the trapping effect of the MR-TADF guest with shallow HOMO energy leve.[6] The direct injection and accumulation of the triplet excitons in the BN-TP site with long lifetime would increase the exciton density and exciton quenching processes are more likely to happen. For the TBP-3MCz-based OLED, TBP-3MCz possesses a slow radiative transition rate (0.80×10^{7} s^{-1}) in compared with the ISC process, which can become the “bottleneck” for exciton utilization in OLED. Such a “trad-off” between radiative transition and RISC process makes realizing efficient TADF with ultra-short excited state lifetime challenging. The introduction of MR-TADF can lead to fast FRET and radiative transition. And the fast FRET process that competes with ISC can reduce the exciton circulation between singlet and triplet states (repeating ISC/RISC process). As a result, the overall excited state lifetime in OLED can be reduced, and both the efficiency and efficiency roll-off of the TBP-3MCz based sensitized device can be improved. Therefore, combining TADF with efficient RISC process and fluorescence guest with fast radiative transition and relieved charge trapping effect can be advantageous for highly efficient roll-off free OLED devices in the future.

Supplementary Table 7. Summary of the device performances of the OLED devices.

Device	V_{on}	λ_{EL} [a)]	L_{max}	CE_{max}	EQE (max/ @ 1000 cd m^{-2}/ @ 3000 cd m^{-2})			CIE [a)]
	V	nm	cd m^{-2}	cd A^{-1}	%			(x, y)
TRZ-3MCz	3.2	505	30683	67.2	24.4	23.8	21.5	(0.21, 0.47)
TRZ-3MCz/ PPF	3.8	512	32750	77.1	26.5	26.5	25.6	(0.22, 0.48)
TB-3MCz	3.4	487	9286	48.1	19.2	14.9	11.2	(0.14, 0.29)
TB-3MCz/ PPF	3.6	486	10432	52.6	23.9	20.6	14.8	(0.14, 0.31)
TBP-3MCz	3.4	521	19823	59.3	19.0	17.0	14.4	(0.27, 0.54)
TBP-3aDMAc	4.2	507	5460	43.1	14.1	7.9	3.7	(0.23, 0.49)
TRZ-3MCz_BN-TP	3.4	525	41343	96.1	24.6	23.1	19.1	(0.23, 0.69)
TB-3MCz_TBN-TPA	3.4	468	8425	34.1	20.2	15.4	9.5	(0.13, 0.19)
TBP -3MCz_BN-TP	3.4	525	30904	89.8	22.0	19.5	16.6	(0.24, 0.68)
TRZ-3MCz_Vac.	3.2	489	32753	68.5	28.2	24.1	21.9	(0.18, 0.40)
TB-3MCz_Vac.	3.0	478	21820	51.0	30.1	26.8	22.3	(0.14, 0.25)

a) EL spectra at current density of 1 mA cm^{-2}

Supplementary Table 8. Comparison of the representative solution-processed OLED device performances with conventional device structures.

TADF emitters	λ_{EL} nm	$EQE_{max/500/1000}$ %	$Roll\text{-}off_{500/1000}$ %	Reference
TRZ-3MCz	505	24.4/24.2/23.8	0.8/2.6	This work
TRZ-3MCz	512	26.5/-/26.5	-	This work
TB-3MCz	487	23.9/23.2/20.6	2.9/13.8	This work
TBP-3MCz	521	19.0/17.4/17.0	8.4/10.5	This work
DCz-DPS-TCz	498	24/21.3/-	11.3/-	*Angew. Chem. Int. Ed.* **2022**, 61, e202115140[7]
tBuCz2m2pTRZ	540	28.7/14.3/-	50.9/-	*Adv. Mater.* **2022**, 34, 2110344[8]
5CzBN-ESF	508	30.6/23.6/20.5	22.9/33.0	*Angew. Chem. Int. Ed.* **2022**, 61, e202212861[9]
YD-TF	552	21.9/-/18.6	-/15.1	*Angew.Chem. Int. Ed.* **2021**, 60, 16585-16593[10]
DMeCzIBN	478	21.6/-/19.6	-/9.3	*Chem. Eng. J.* **2021**, 412, 128574.[11]
T-CNDF-T-tCz	484	21.0/-/-	-/-	*Adv. Sci.* **2020**, 7, 1902087.[12]
OAB-ABP-1	506	19.6/-/17.4	-/11.2	*Adv. Mater.* **2020**, 2004072[13]
TBP-DMAc	526	22.1/-/20.3	-/8.1	*Adv. Funct. Mater.* **2018**, 28, 1704927[14]

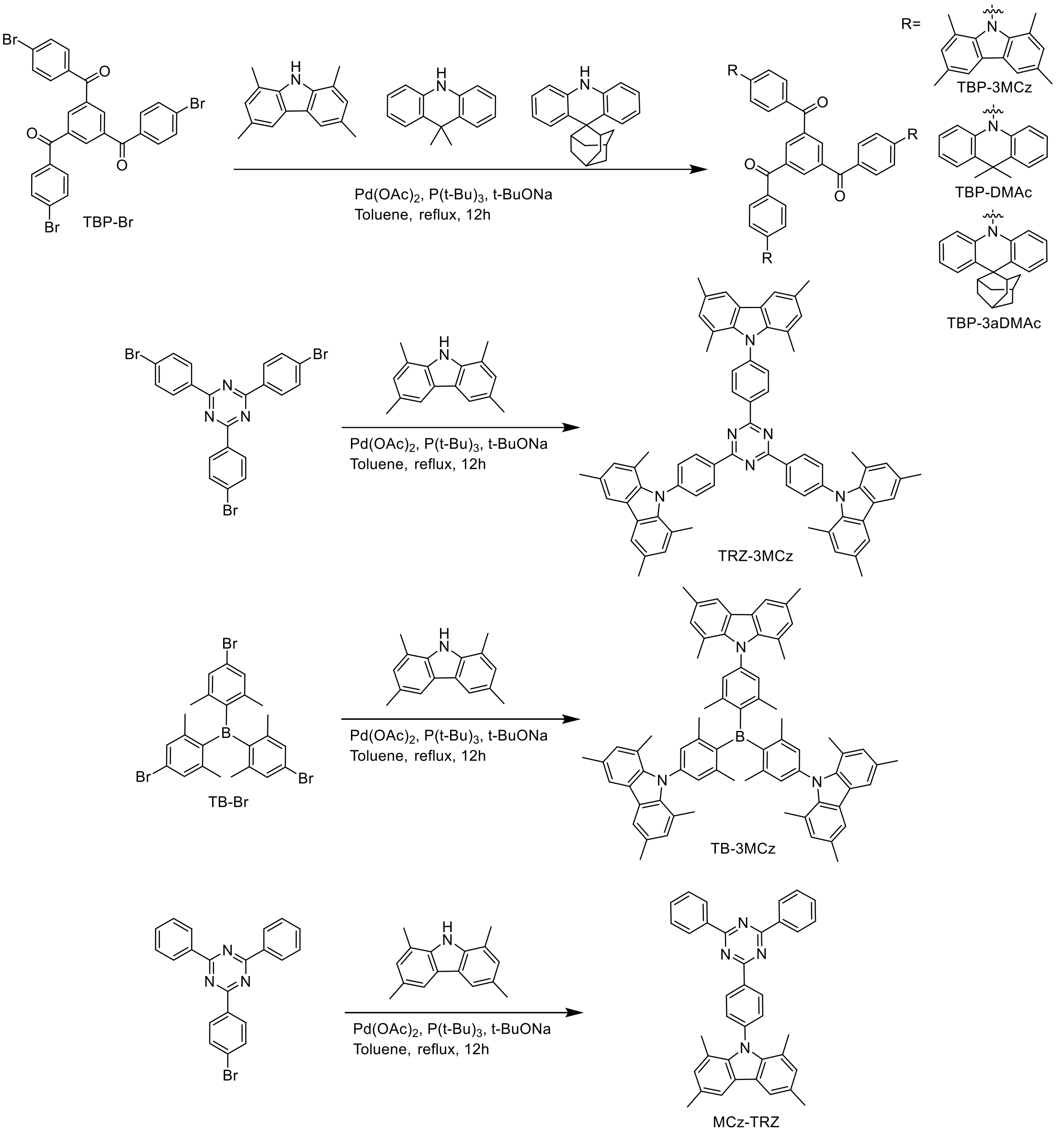

Supplementary Fig 27. Synthetic routes of the investigated compounds.

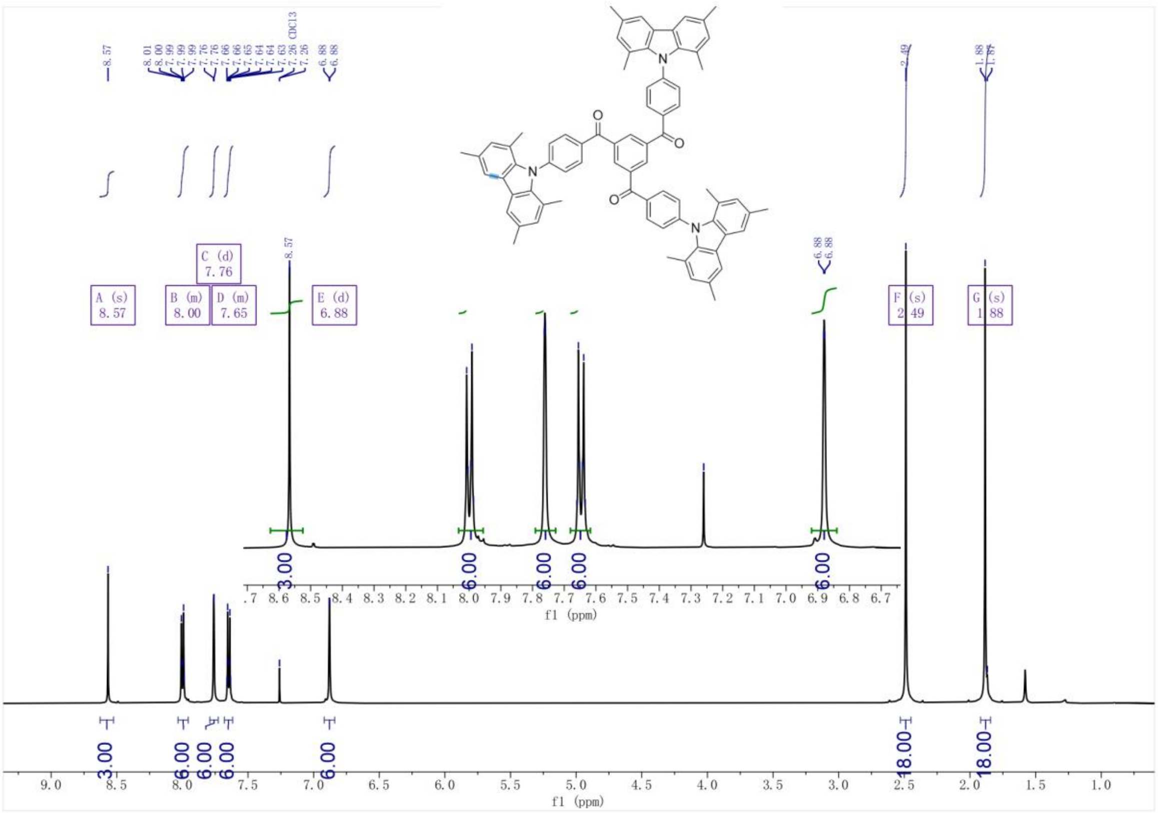

Supplementary Fig. 28 ^{1}H NMR spectrum of TBP-3MCz in $CDCl_3$.

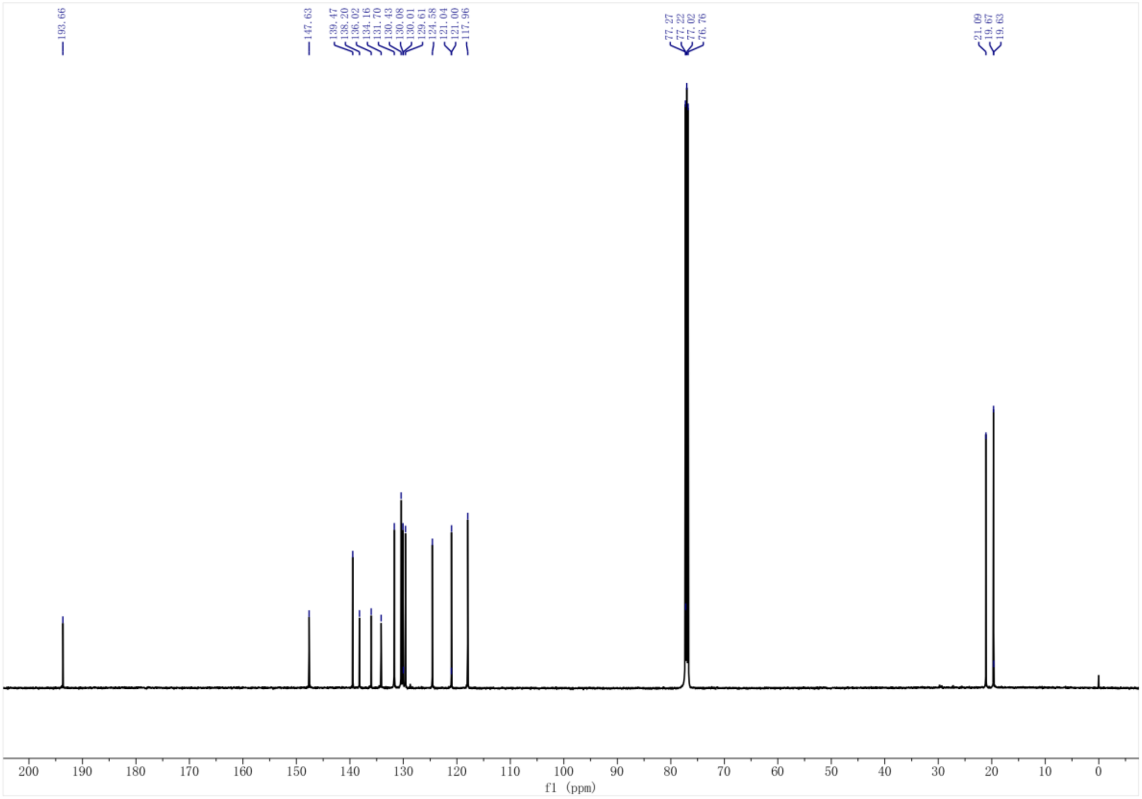

Supplementary Fig. 29 ^{13}C NMR spectra of TBP-3MCz in $CDCl_3$.

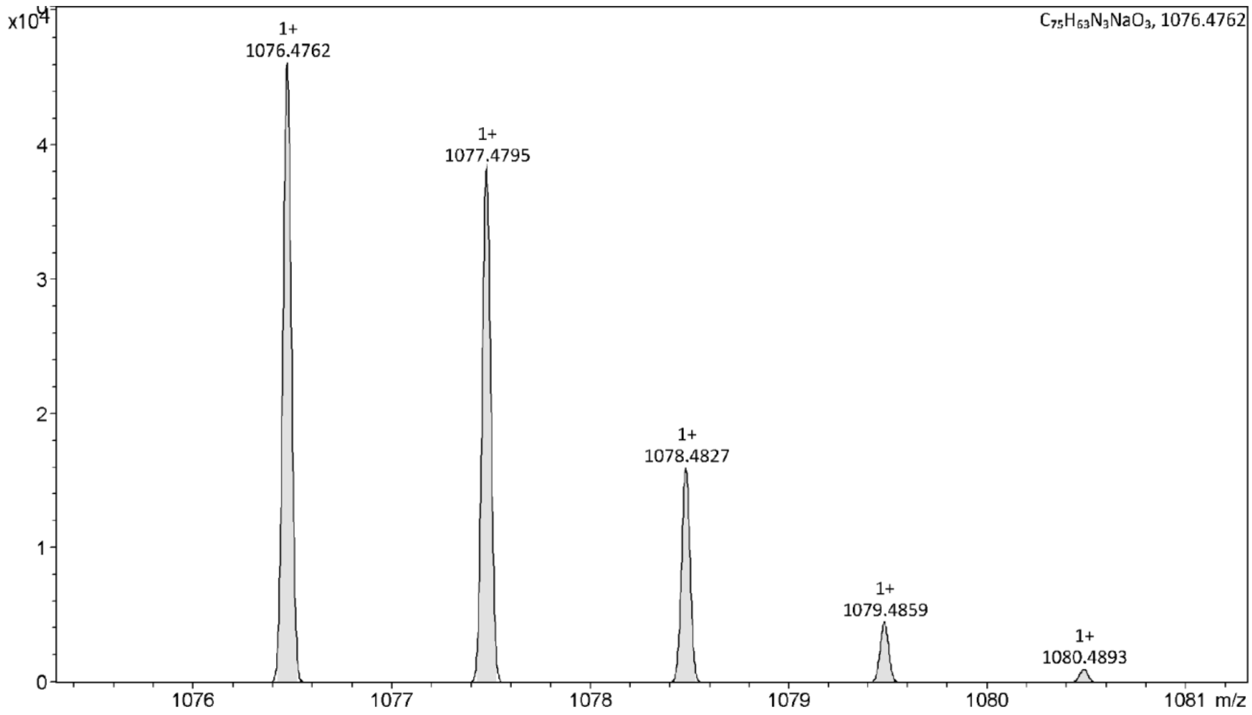

Supplementary Fig. 30 High-resolution mass spectrometry of TBP-3MCz.

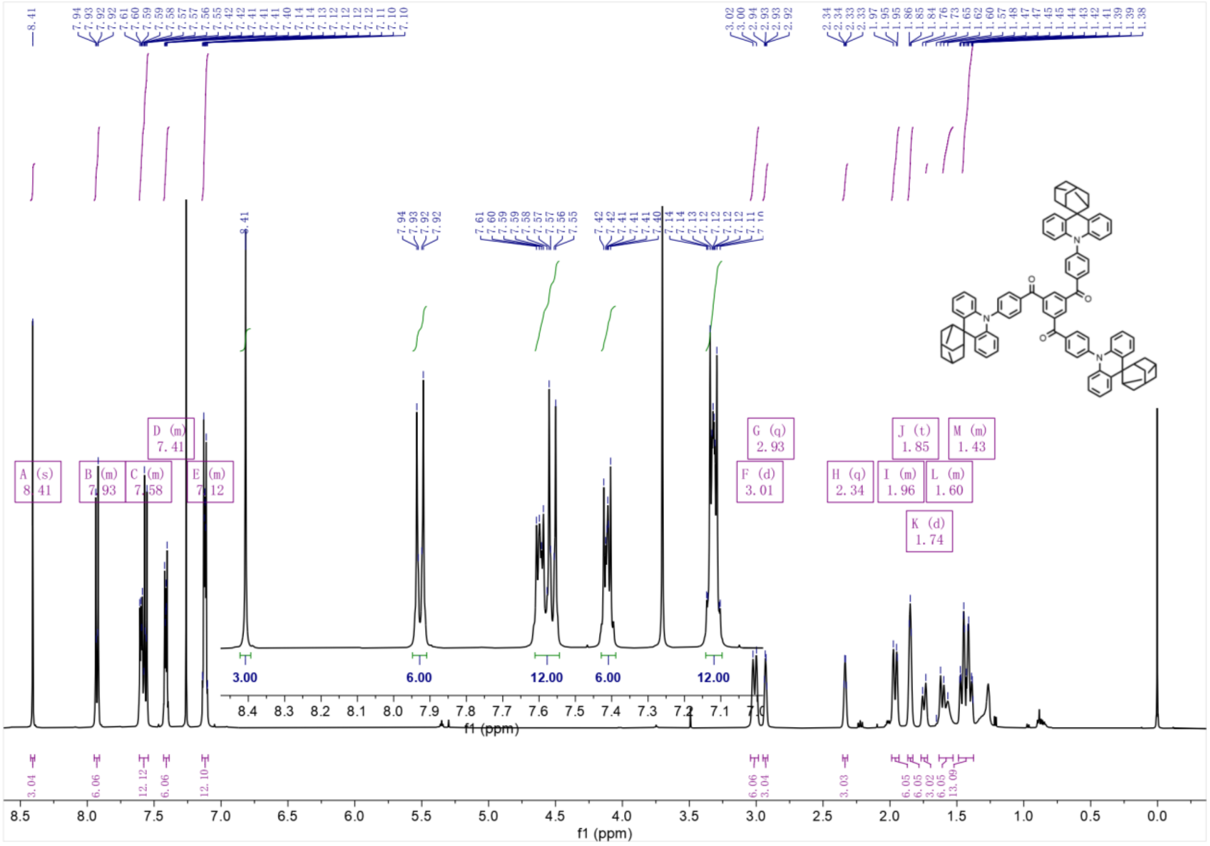

Supplementary Fig. 31 ^{1}H NMR spectrum of TBP-3aDMAc in $CDCl_3$.

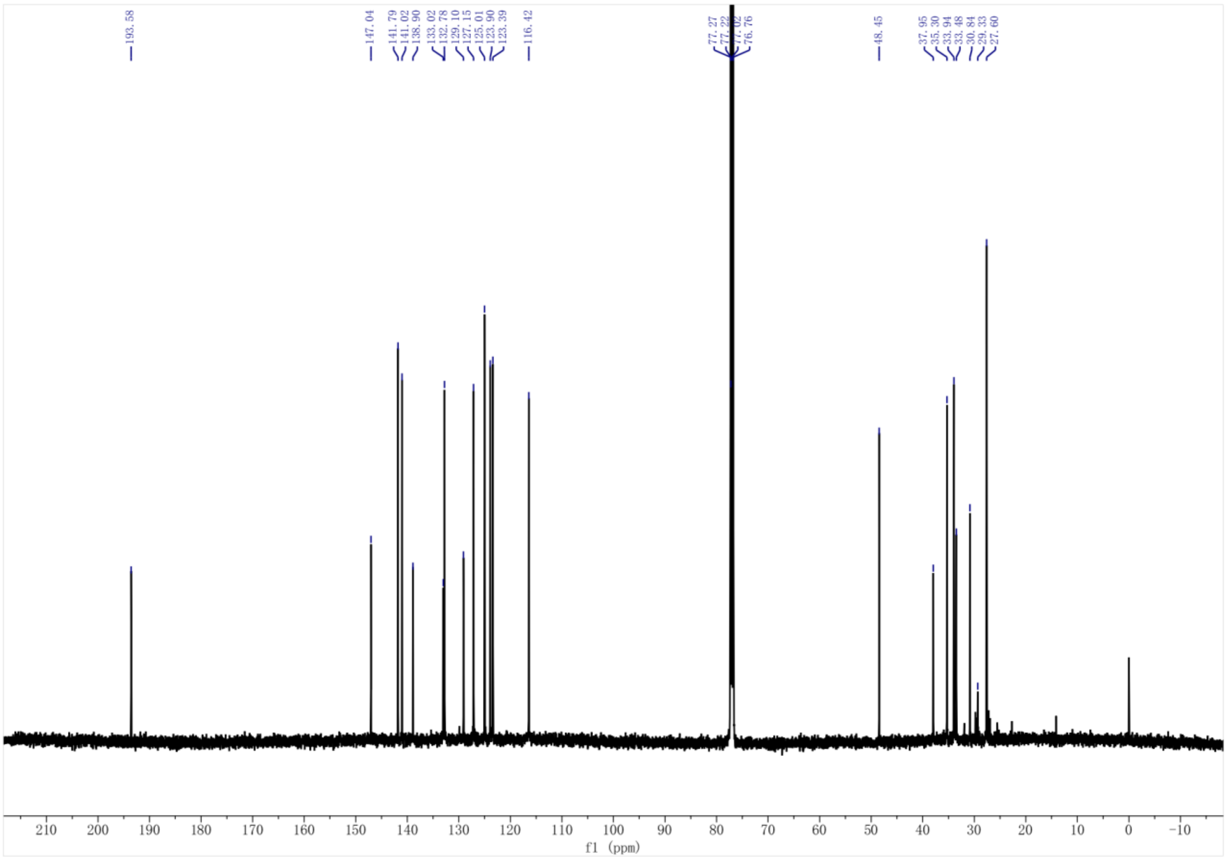

Supplementary Fig. 32 ^{13}C NMR spectrum of TBP-3aDMAc in $CDCl_3$.

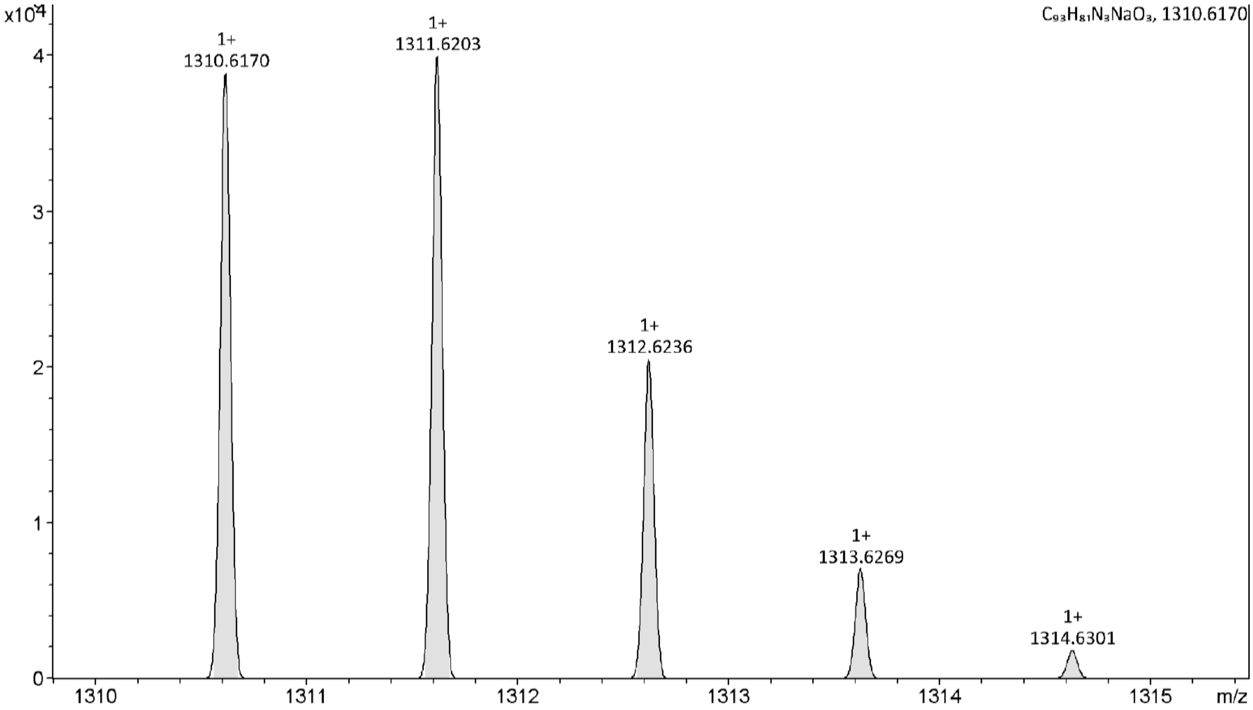

Supplementary Fig. 33 High-resolution mass spectrometry of TBP-3aDMAc.

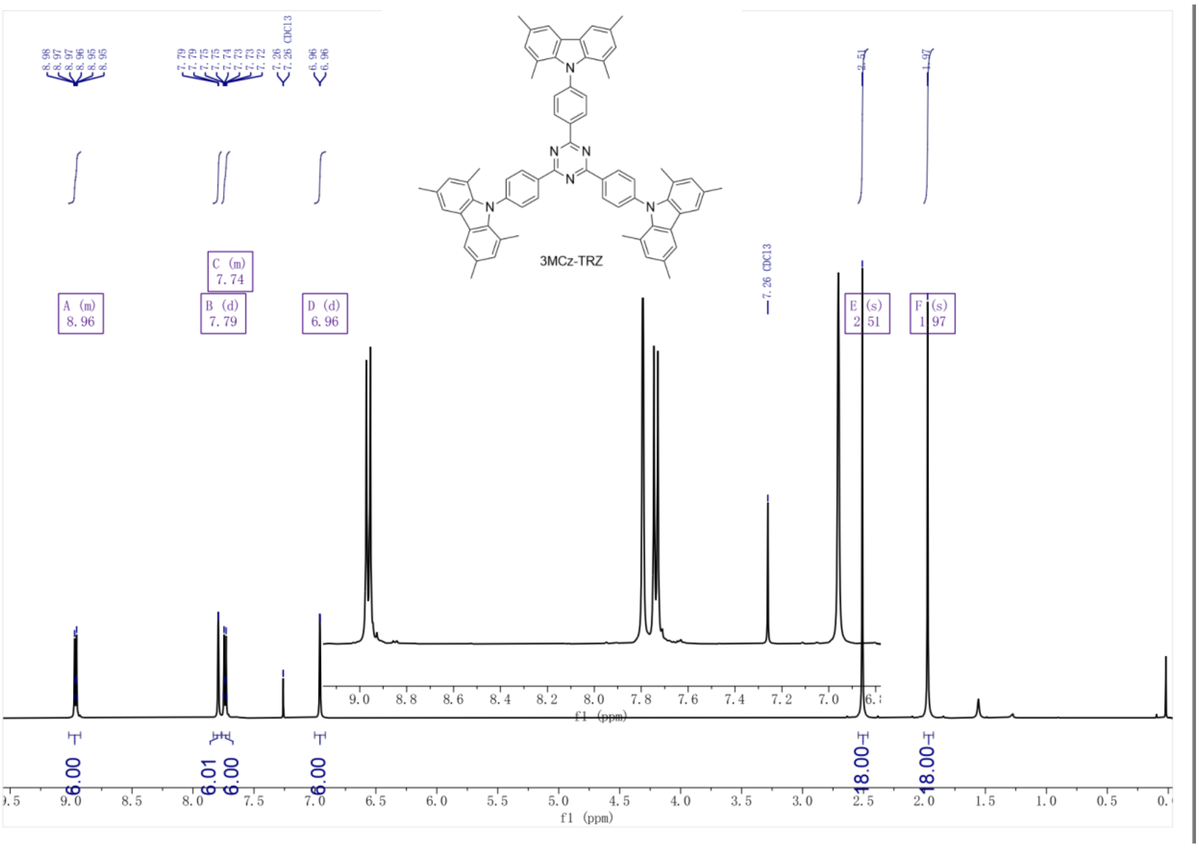

Supplementary Fig. 34 ^{1}H NMR spectrum of TRZ-3MCz in $CDCl_3$.

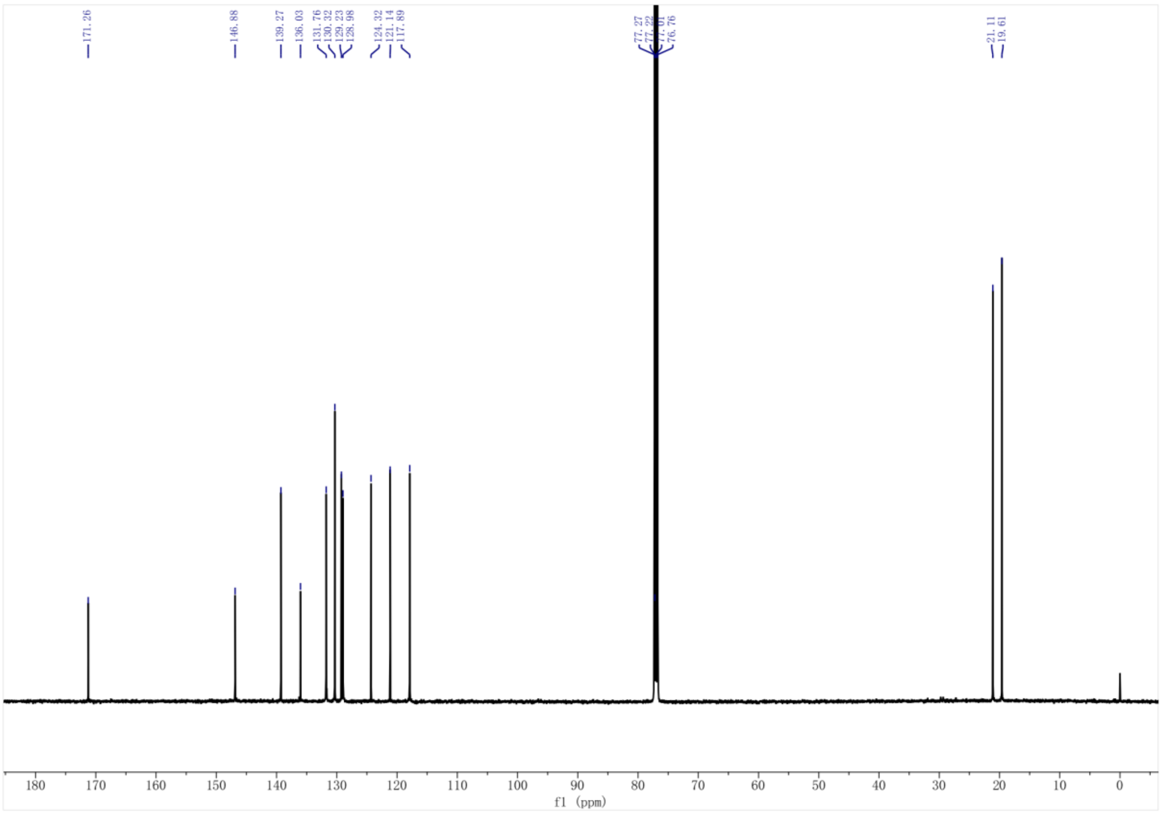

Supplementary Fig. 35 ^{13}C NMR spectrum of TRZ-3MCz in $CDCl_3$.

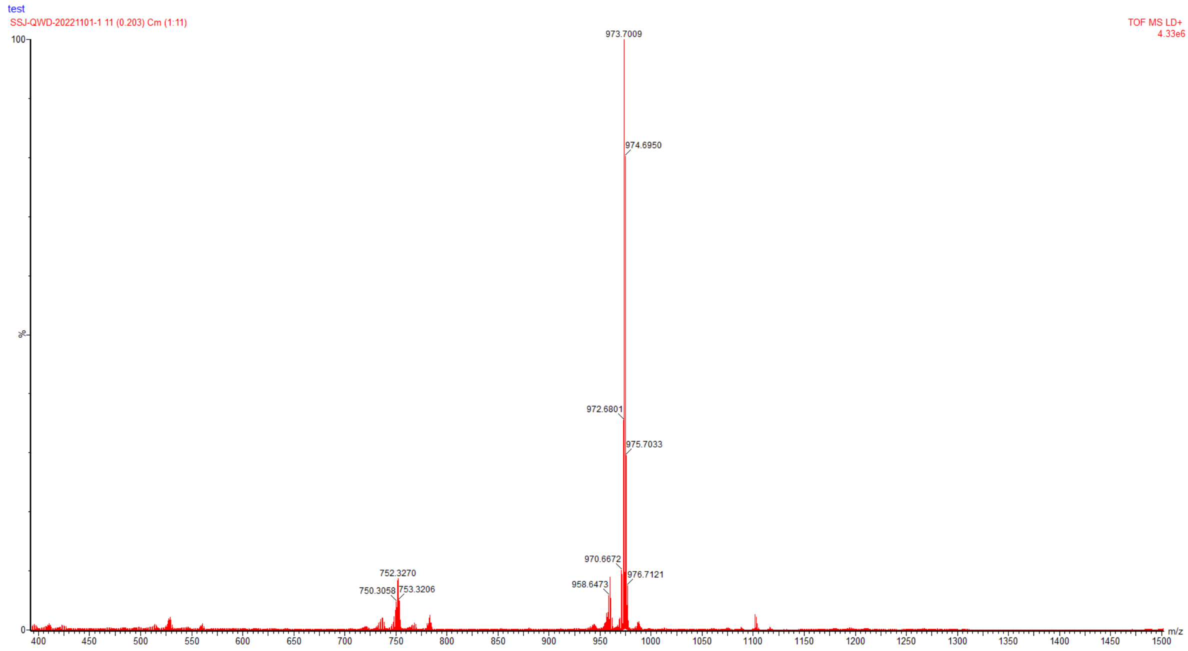

Supplementary Fig. 36 MALDI-TOF mass spectrometry of TRZ-3MCz.

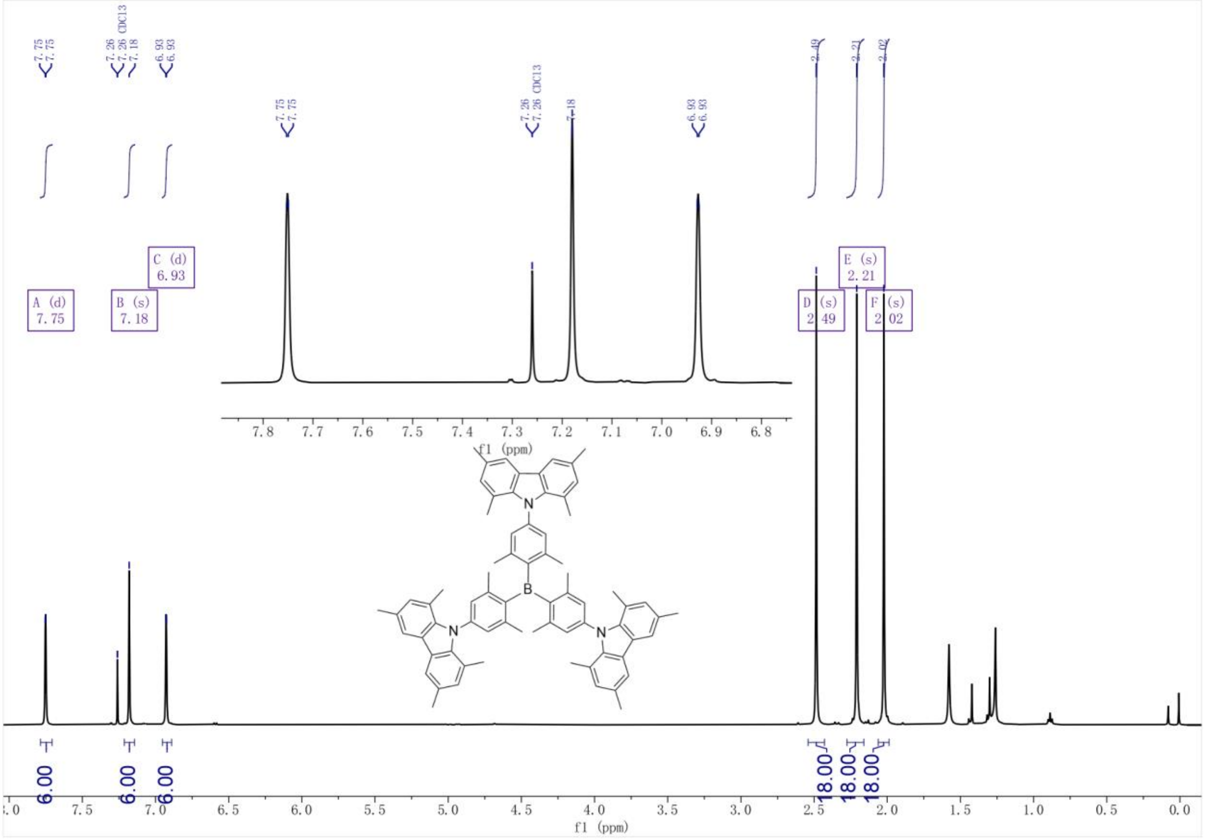

Supplementary Fig. 37 ^{1}H NMR spectrum of TB-3MCz in $CDCl_3$.

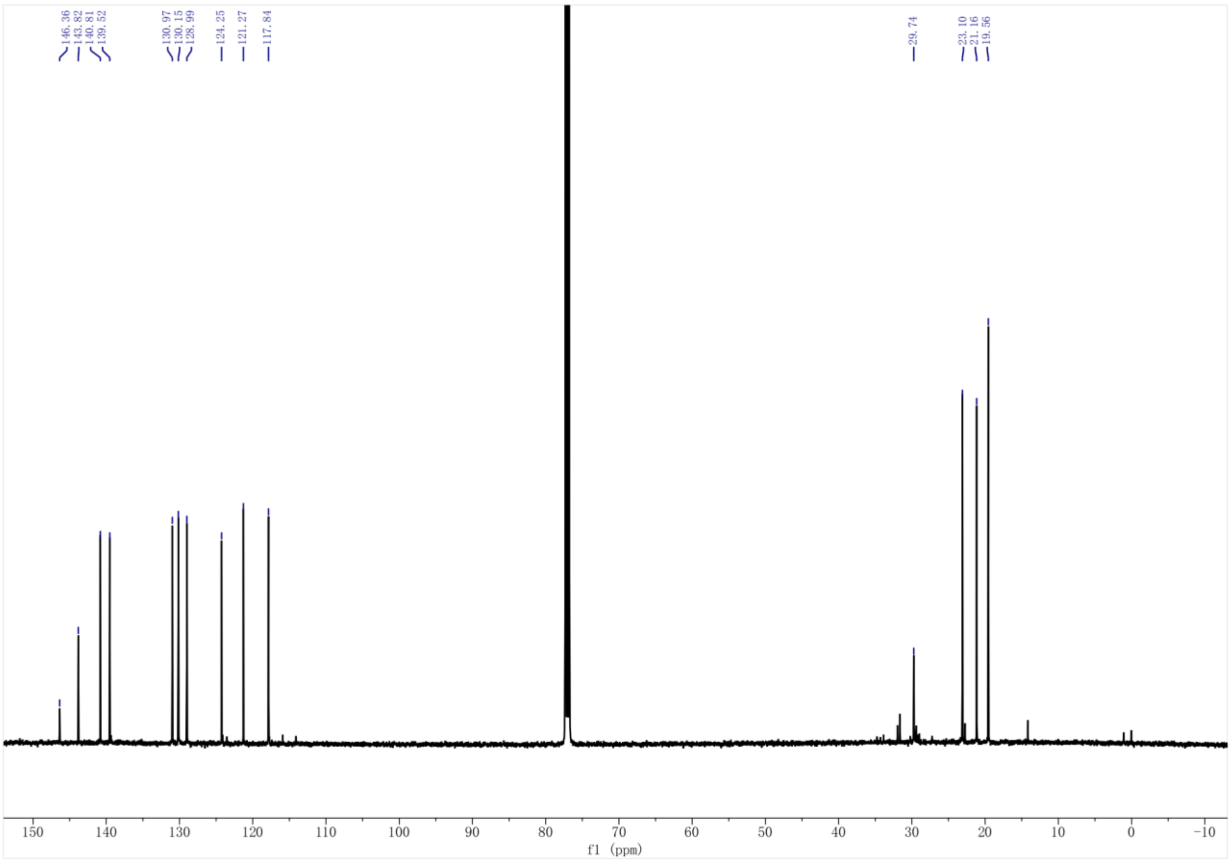

Supplementary Fig. 38 ^{13}C NMR spectrum of TB-3MCz in $CDCl_3$.

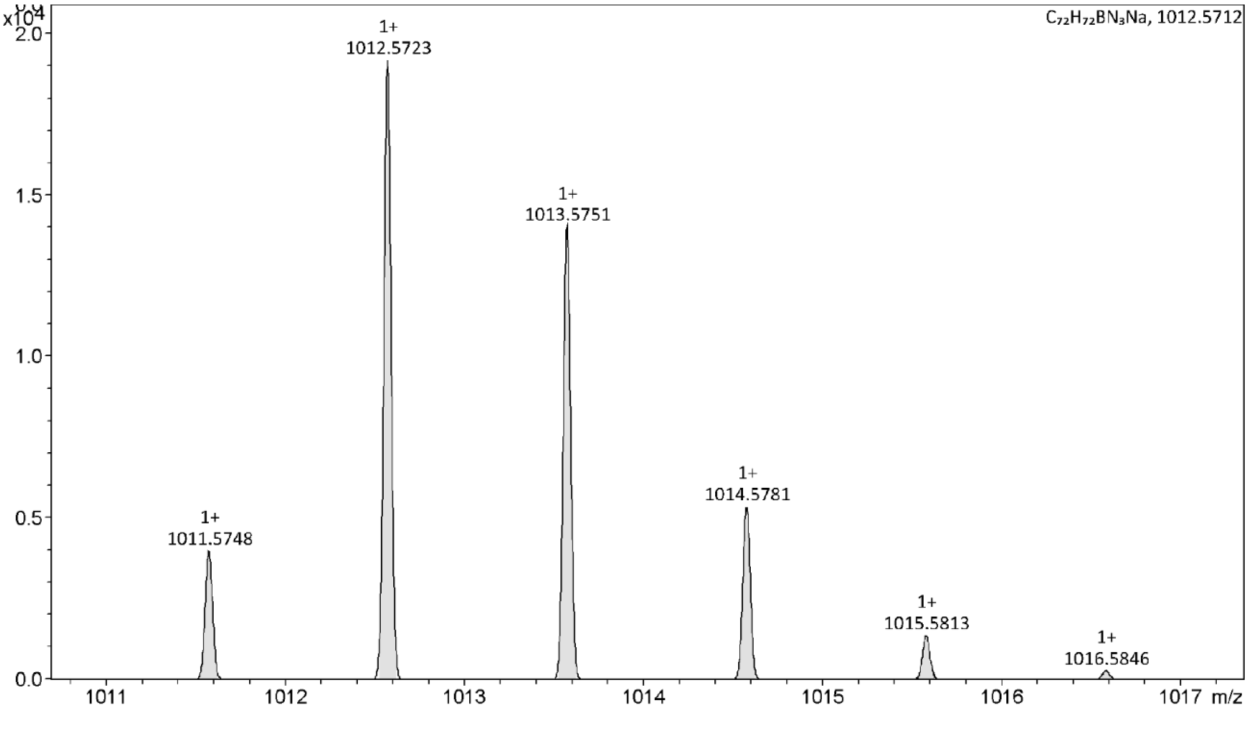

Supplementary Fig. 39 High-resolution mass spectrometry of TB-3MCz.

Supplementary References

1 Goushi, K., Yoshida, K., Sato, K. & Adachi, C. Organic light-emitting diodes employing efficient reverse intersystem crossing for triplet-to-singlet state conversion. *Nat. Photonics* **6**, 253-258 (2012).

2 Cui, L. S. *et al.* Fast spin-flip enables efficient and stable organic electroluminescence from charge-transfer states. *Nat. Photonics* **14**, 636-642 (2020).

3 Wada, Y., Nakagawa, H., Matsumoto, S., Wakisaka, Y. & Kaji, H. Organic light emitters exhibiting very fast reverse intersystem crossing. *Nat. Photonics* **14**, 643–649 (2020).

4 Kelly, D., Franca, L. G., Stavrou, K., Danos, A. & Monkman, A. P. Laplace transform fitting as a tool to uncover distributions of reverse intersystem crossing rates in tadf systems. *J. Phys. Chem. Lett.* **13**, 6981-6986 (2022).

5 Schmidt, T. D. *et al.* Emitter orientation as a key parameter in organic light-emitting diodes. *Phys. Rev. Applied* **8**, 037001 (2017).

6 Kim, E. *et al.* Highly efficient and stable deep-blue organic light-emitting diode using phosphor-sensitized thermally activated delayed fluorescence. *Sci. Adv.* **8**, eabq1641 (2022).

7 Li, C. *et al.* Asymmetrical-dendronized TADF emitters for efficient non-doped solution-processed oleds by eliminating degenerate excited states and creating solely thermal equilibrium routes. *Angew. Chem. Int. Ed.* **61**, e202115140 (2022).

8 Sun, D. *et al.* Thermally activated delayed fluorescent dendrimers that underpin high-efficiency host-free solution-processed organic light-emitting diodes. *Adv. Mater.* **34**, e2110344 (2022).

9 Zhao, G. *et al.* Exceeding 30 % external quantum efficiency in non-doped OLEDs utilizing solution processable TADF emitters with high horizontal dipole orientation via anchoring strategy. *Angew. Chem. Int. Ed.* **61**, e202212861 (2022).

10 Wang, X. *et al.* π-stacked donor-acceptor dendrimers for highly efficient white electroluminescence. *Angew. Chem. Int. Ed.* **60**, 16585-16593 (2021).

11 Kreiza, G. *et al.* High efficiency and extremely low roll-off solution- and vacuum-processed OLEDs based on isophthalonitrile blue TADF emitter. *Chem. Eng. J.* **412**, 128574 (2021).

12 Zheng, X. *et al.* Achieving 21% external quantum efficiency for nondoped solution-processed sky-blue thermally activated delayed fluorescence OLEDs by means of multi-(donor/acceptor) emitter with through-space/-bond charge transfer. *Adv. Sci.* **7**, 1902087 (2020).

13 Ikeda, N. *et al.* Solution-processable pure green thermally activated delayed fluorescence emitter based on the multiple resonance effect. *Adv. Mater.*, e2004072 (2020).

14 Cai, X. Y. *et al.* "Trade-off" hidden in condensed state solvation: Multiradiative channels design for highly efficient solution-processed purely organic electroluminescence at high brightness. *Adv. Funct. Mater.* **28**, 1704927 (2018).
