# Supplementary material for: Confining donor conformation distributions for efficient thermally activated delayed fluorescence with fast spin-flipping
Source: Nat Commun. 2023 May 4;14:2564. doi: 10.1038/s41467-023-38197-y (PMC10160101; doi:10.1038/s41467-023-38197-y)

checkCIF/PLATON report

Structure factors have been supplied for datablock(s) 1

THIS REPORT IS FOR GUIDANCE ONLY. IF USED AS PART OF A REVIEW PROCEDURE FOR PUBLICATION, IT SHOULD NOT REPLACE THE EXPERTISE OF AN EXPERIENCED CRYSTALLOGRAPHIC REFEREE.

No syntax errors found. CIF dictionary Interpreting this report

Datablock: 1

```
Bond precision:   C-C = 0.0019 A          Wavelength=1.54184

Cell:              a=21.8186(4)     b=21.8186(4)     c=25.1998(5)
                   alpha=90         beta=90          gamma=120
Temperature:       150 K

                Calculated                  Reported
Volume          10389.2(4)                  10389.2(4)
Space group     R -3                        R -3
Hall group      -R 3                        -R 3
Moiety formula  C93 H81 N3 O3 [+ solvent]   C31 H27 N O
Sum formula     C93 H81 N3 O3 [+ solvent]   C18.33 H16.67 N1.67 O1.67
Mr              1288.61                     287.00
Dx,g cm-3       1.236                       0.826
Z               6                           18
Mu (mm-1)       0.569                       0.425
F000            4104.0                      2730.0
F000'           4114.97
h,k,lmax        26,26,30                    26,26,30
Nref            4117                        4121
Tmin,Tmax       0.960,0.967                 0.887,1.000
Tmin'           0.934

Correction method= # Reported T Limits: Tmin=0.887 Tmax=1.000
AbsCorr = MULTI-SCAN

Data completeness= 1.001           Theta(max)= 67.027

R(reflections)= 0.0379( 3940)                          wR2(reflections)=
                                                       0.1005( 4121)
S = 1.032                  Npar= 298
```

The following ALERTS were generated. Each ALERT has the format

test-name_ALERT_alert-type_alert-level.

Click on the hyperlinks for more details of the test.

Alert level C

```
PLAT230_ALERT_2_C Hirshfeld Test Diff for    C00L     --C00N     .         5.2 s.u.
PLAT410_ALERT_2_C Short Intra H...H Contact  H00K     ..H00X     .        1.99 Ang.
                                                      x,y,z  =      1_555 Check
PLAT410_ALERT_2_C Short Intra H...H Contact  H00Q     ..H00S     .        1.99 Ang.
                                                      x,y,z  =      1_555 Check
```

Alert level G

```
FORMU01_ALERT_1_G  There is a discrepancy between the atom counts in the
            _chemical_formula_sum and _chemical_formula_moiety. This is
            usually due to the moiety formula being in the wrong format.
            Atom count from _chemical_formula_sum:   C18.33 H16.67 N1.67 O1.67
            Atom count from _chemical_formula_moiety:C31 H27 N1 O1
FORMU01_ALERT_2_G  There is a discrepancy between the atom counts in the
            _chemical_formula_sum and the formula from the _atom_site* data.
            Atom count from _chemical_formula_sum:C18.33 H16.67 N1.67 O1.67
            Atom count from the _atom_site data:  C31 H27 N1 O1
CELLZ01_ALERT_1_G Difference between formula and atom_site contents detected.
CELLZ01_ALERT_1_G ALERT: Large difference may be due to a
            symmetry error - see SYMMG tests
           From the CIF: _cell_formula_units_Z   18
           From the CIF: _chemical_formula_sum  C18.33 H16.67 N1.67 O1.67
           TEST: Compare cell contents of formula and atom_site data

           atom    Z*formula  cif sites diff
           C        329.94    558.00 -228.06
           H        300.06    486.00 -185.94
           N         30.06     18.00   12.06
           O         30.06     18.00   12.06
PLAT041_ALERT_1_G Calc. and Reported SumFormula    Strings  Differ     Please Check
PLAT042_ALERT_1_G Calc. and Reported MoietyFormula Strings  Differ     Please Check
PLAT045_ALERT_1_G Calculated and Reported Z Differ by a Factor ...      0.333 Check
PLAT051_ALERT_1_G Mu(calc) and Mu(CIF) Ratio Differs from 1.0 by .      33.78 %
PLAT083_ALERT_2_G SHELXL Second Parameter in WGHT  Unusually Large       9.47 Why ?
PLAT605_ALERT_4_G Largest Solvent Accessible VOID in the Structure        283 A**3
PLAT720_ALERT_4_G Number of Unusual/Non-Standard Labels ..........         60 Note
PLAT909_ALERT_3_G Percentage of I>2sig(I) Data at Theta(Max) Still        89% Note
PLAT978_ALERT_2_G Number C-C Bonds with Positive Residual Density.          6 Info
```

0 **ALERT level A** = Most likely a serious problem - resolve or explain
0 **ALERT level B** = A potentially serious problem, consider carefully
3 **ALERT level C** = Check. Ensure it is not caused by an omission or oversight
13 **ALERT level G** = General information/check it is not something unexpected

7 ALERT type 1 CIF construction/syntax error, inconsistent or missing data
6 ALERT type 2 Indicator that the structure model may be wrong or deficient
1 ALERT type 3 Indicator that the structure quality may be low
2 ALERT type 4 Improvement, methodology, query or suggestion
0 ALERT type 5 Informative message, check

It is advisable to attempt to resolve as many as possible of the alerts in all categories. Often the minor alerts point to easily fixed oversights, errors and omissions in your CIF or refinement strategy, so attention to these fine details can be worthwhile. In order to resolve some of the more serious problems it may be necessary to carry out additional measurements or structure refinements. However, the purpose of your study may justify the reported deviations and the more serious of these should normally be commented upon in the discussion or experimental section of a paper or in the "special_details" fields of the CIF. checkCIF was carefully designed to identify outliers and unusual parameters, but every test has its limitations and alerts that are not important in a particular case may appear. Conversely, the absence of alerts does not guarantee there are no aspects of the results needing attention. It is up to the individual to critically assess their own results and, if necessary, seek expert advice.

Publication of your CIF in IUCr journals

A basic structural check has been run on your CIF. These basic checks will be run on all CIFs submitted for publication in IUCr journals (*Acta Crystallographica*, *Journal of Applied Crystallography*, *Journal of Synchrotron Radiation*); however, if you intend to submit to *Acta Crystallographica Section C* or *E* or *IUCrData*, you should make sure that full publication checks are run on the final version of your CIF prior to submission.

Publication of your CIF in other journals

Please refer to the *Notes for Authors* of the relevant journal for any special instructions relating to CIF submission.

PLATON version of 18/05/2022; check.def file version of 17/05/2022

Datablock 1 - ellipsoid plot

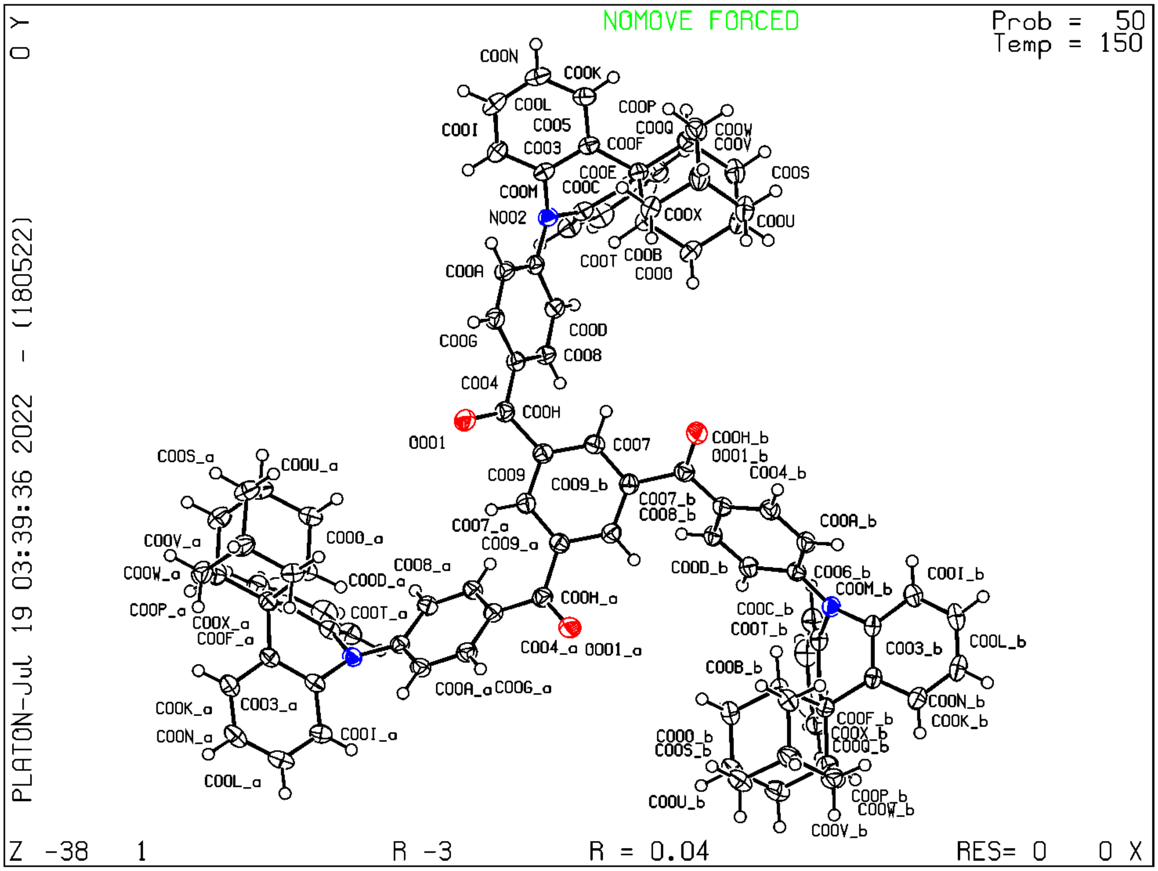

Supplement: Supplementary file 3 — Source Data [file 41467_2023_38197_MOESM3_ESM.zip › TBP-3aDMAc checkcif.pdf]
